# No evidence of resistance to itraconazole in a prospective real-world trial of dermatomycosis in India

**DOI:** 10.1371/journal.pone.0281514

**Published:** 2023-02-14

**Authors:** S. Handa, A. Villasis-Keever, M. Shenoy, S. Anandan, M. Bhrushundi, N. Garodia, D. Fife, P. De Doncker, K. Shalayda, P. Hu, S. Fonseca, N. Cure-bolt

**Affiliations:** 1 Postgraduate Institute of Medical Education & Research, Chandigarh, India; 2 Janssen Research & Development, LLC, Titusville, New Jersey, United States of America; 3 Yenepoya Medical College Hospital, Mangalore, Karnataka, India; 4 Sri Ramchandra Hospital, Chennai, Tamil Nadu, India; 5 Lata Mangeshkar Hospital, Nagpur, Maharashtra, India; 6 Janssen Medical Affairs, Mumbai, Maharashtra, India; 7 Janssen Infectious Diseases-Diagnostics, Beerse, Belgium; 8 Janssen Research & Development, LLC, Raritan, New Jersey, United States of America; Gulu University, UGANDA

## Abstract

**Background:**

The prevalence of superficial fungal infections in India is believed to have increased substantially in the past decade. We evaluated the treatment outcomes and risk factors associated with clinical response to a treatment course of itraconazole for the management of dermatomycosis in India.

**Methods:**

In this real-world, prospective pilot study (August 2019 to March 2020), adult participants (18–60 years), diagnosed with *T*. *cruris* or *T*. *corporis*, received itraconazole 200 mg/day (any formulation) orally for 7 days, and were followed for an additional 7 days.

**Results:**

The study was terminated early due to the COVID-19 pandemic. Of 40 enrolled participants (mean [SD] age, 35.5 [12.73] years; {62.5%}] male; 37 received itraconazole and 20 (50%) completed the study. The median (range) Clinical Evaluation Tool Signs and Symptoms total score at baseline was 5.5 (2–10). Clinical response of “healed” or “markedly improved” based on the Investigator Global Evaluation Tool at day 7 (primary objective) was 42.9% (12/28; 95% CI: 24.53%, 61.19%). Itraconazole minimum inhibitory concentration for identified microorganisms, *T*. *mentagrophytes* species complex (91.7%) and *T*. *rubrum* (8.3%), was within the susceptibility range (0.015–0.25 mcg/mL). At day 14, 8/13 (61.5%) participants achieved a mycological response, 2/13 participants (15.4%) had a mycological failure and 90% showed a clinical response.

**Conclusion:**

COVID-19 pandemic affected patient recruitment and follow-up, so the findings call for a careful interpretation. Nevertheless, this real-world study reconfirmed the clinical efficacy and microbial susceptibility to itraconazole for the fungi causing dermatophytosis in India.

**Trial registration:**

Trial registration number: Clinicaltrials.gov NCT03923010.

## Introduction

Superficial cutaneous fungal infections or dermatomycosis/dermatophytosis are one of the most common dermatoses affecting around 20–25% of the world population [1]. India has a tropical-like climate with a high prevalence of fungal infections and marked variation reported across the country ranging from 36.6–78.4% [2]. Although nationwide data is lacking, the prevalence and severity of the disease have increased at an alarming rate over the last decade as per physician perception based on the number of cases seen in their outpatient departments. There are also anecdotal reports of a lack of efficacy with traditionally used topical and systemic antifungals [3–6]. This observed increase in frequency and severity has been attributed to many factors including the widespread use of high-potency steroids in topical formulations and misuse of antifungal agents. Host-related factors, abnormal drug pharmacokinetics (PK) [7, 8], and differences in bioavailability among many itraconazole formulations available in India [9], have been considered among the factors for treatment failures. To date, there is no clear evidence that supports these observations.

The clinical presentation of dermatomycosis varies from mild scaling and redness to severe inflammation [10–13]. Although painless and superficial, these dermatophyte infections have a significant negative impact on the social, psychological, and occupational life of the individual and can lead to emotional and financial distress [12, 14–16]. The management armamentarium for treatment of the disease includes topical and oral antifungals. Several newer azoles, including efinaconazole, luliconazole, sertaconazole, oxaborole, and tavaborole, have either been introduced or are being studied for topical use [17]. Oral fluconazole, griseofulvin, itraconazole and terbinafine have been used extensively. Itraconazole has remained an important treatment choice for superficial fungal infections and has shown its clinical value in India due to increasing resistance to other antifungals [18–20], atypical and unusual presentations of dermatophytosis [21] and the *Trichophyton mentagrophytes* pandemic in recent years [4, 22]. Cases of treatment failures have also been reported with itraconazole, though not linked to anti-fungal resistance [8, 20, 23].

Itraconazole, a triazole antifungal agent, has a broad spectrum of activity against infections caused by dermatophytes, yeasts, and many other fungi [24–27]. Itraconazole and its main metabolite, hydroxy-itraconazole both have a prominent affinity to fungal cytochrome P-450 (CYP), which is involved in the biosynthesis of ergosterol from lanosterol [28, 29]. Ergosterol is a vital cell membrane component in fungi [30] and its specific inhibition by itraconazole results in drastic inhibition of fungal growth [7].

The objective of this study was to evaluate real-world treatment outcomes and the risk factors associated with a poor clinical response with the use of a of itraconazole in patients with dermatomycosis.

## Methods

### Study design

This real-world, prospective, non-randomized, multicenter, interventional, longitudinal study was conducted in India and consisted of an open-label treatment phase of 7 days followed by a follow-up phase of 7 days.

The primary objective of the study was to estimate the proportion of participants that received itraconazole for *Tinea cruris* (*T*. *cruris*) or *Tinea corporis* (*T*. *corporis*) who demonstrated clinical response after 7 days of treatment. The secondary objectives were to estimate the proportion of participants with the mycological response after 14 days of follow-up and the association of clinical response with plasma drug concentrations (itraconazole and hydroxy-itraconazole) and baseline sensitivity pattern of causative fungi. Other exploratory objectives included the estimation of the proportion of participants with the clinical response after 14 days of follow-up and the extent to which clinical improvement at Day 7 predicts clinical improvement at Day 14.

The study is registered with clinicaltrials.gov (NCT03923010). The study protocol, informed consent forms, and applicable study documents were approved by an independent ethics committee or institutional review board at each study site. They include Institutional Ethics Committee, NKP Salve Institute of Medical Science & Lata Mangeshkar Hospital, Nagpur, India; Institutional Ethics Committee, Postgraduate Institute of Medical Education and Research, Chandigarh, India; Institutional Ethics Committee, Sri Ramachandra Hospital, Chennai, India and Yenepoya Ethics Committee-1, University Road, Deralakatte, Mangalore, India. Signed informed consent was obtained from all participants at the start of the study. This study was conducted in accordance with the Declaration of Helsinki and is consistent with Good Clinical Practices and applicable regulatory requirements. The planned recruitment time for this study was 4 months. However, the period was extended to 10 months due to the Coronavirus Disease-2019 (COVID-19) pandemic. The study was eventually discontinued prematurely for the same reason.

### Study population

Adult participants aged ≥18 to ≤60 years with a clinical diagnosis of *T*. *cruris* or *T*. *corporis* infection, with or without a history of antifungal treatment were enrolled if they were prescribed oral itraconazole 200 mg/day for the management of their dermatophytosis. The participants were recruited from clinical practices of dermatologists from four sites (Nagpur, Chandigarh, Chennai, and Mangalore) in India. Exclusion criteria included the history of ventricular dysfunction, liver or renal insufficiency; contraindications for the use of itraconazole; known achlorhydria or treatment for gastric acidity; the presence of other dermatoses e.g., psoriasis, seborrheic, or atopic dermatitis or infection with an organism with known or established resistance to itraconazole; co-existing fungal infection of other body areas, known allergies or receipt of CYP3A4 substrates or oral itraconazole within 14 days or systemic antifungal or corticosteroid therapy within 30 days or a topical antifungal or corticosteroid therapy within 14 days before screening.

### Treatment

Participants were prescribed locally available itraconazole (either generic or reference). The dosage was a 200 mg capsule or 2 capsules of 100 mg each, once daily. Participants were instructed to take their prescribed dose of itraconazole orally each day after a full meal at home. The recommended duration of treatment was 7 days. The total duration was decided by the treating physician as part of the participant’s clinical care, according to local standards of practice, and could be extended up to Day 14.

### Assessments

Participants’ demographic information was collected at baseline. The clinical assessment of the disease was evaluated by the treating physicians using two tools. The Clinical Evaluation Tool Signs and Symptoms (CET SS; total score ranges from 0 to 18) was used in the development program of itraconazole. It evaluates the presence and severity (absent = 0, mild = 1, moderate = 2, severe = 3) of each of 6 signs and symptoms (desquamation; erythema and pruritus; exudation or incrustation; infiltration; maceration; vesiculation or pustules). The Investigator Global Evaluation (IGE) tool was used to classify the clinical response based on the percentage of clinical improvement from baseline. The CET SS total scores at Day 7 and Day 14 were compared with baseline scores to assess the percentage of clinical improvement, defined as a decrease in CET SS total score to 1 or 2, which was further used to classify the clinical efficacy using the Investigator Global Evaluation (IGE) tool, with scores 1 (absence of signs and symptoms) or 2 (≥ 50% clinical improvement). Clinical response was thus defined as having scores 1 or 2 (considered as “healed” or “markedly improved”) on the IGE tool of clinical improvement while the mycological response was defined as culture negative at Day 14 from participants positive at baseline.

Pharmacokinetic (PK) assessments were conducted in participants who had at least 1 set of blood samples drawn at Day 7. Measurement of itraconazole and hydroxyitraconazole concentrations in plasma was performed using high performance liquid chromatography with tandem mass spectrometry. Blood samples for PK analysis were collected predose (at 24 hours (±2 hours) after the previous dose on Day 6) and Day 13 and postdose (at 2 hours and 4.5 hours) after the Day 7 and Day 14 doses. Plasma concentrations of itraconazole and hydroxy-itraconazole were assessed against clinical outcomes.

Skin scrapings were collected for culture and sensitivity analysis and the baseline resistance to itraconazole and its association with clinical outcomes was evaluated. Skin specimens were collected from the edge of lesions using sterile blunt scalpel. The KOH samples were analyzed locally at each study center and for mycological culture analysis samples were shipped to central laboratory (University of Texas—Fungus Testing Lab—San Antonio, Texas). The fungal pathogens were identified by amplification and Sanger-based (dideoxy-termination) sequencing as per the Clinical and Laboratory Standards Institute (CLSI) MM18-A guidelines. A microdilution method, according to the Clinical and Laboratory Standards Institute (CLSI) M27 guidelines, was used to conduct antifungal sensitivity testing [31]. The minimum inhibitory concentration (MIC) of itraconazole of fungal pathogens from each patient was determined from the baseline samples, which had been taken before the administration of the study agent. Further details on culture and sensitivity analysis are provided in the S4 File.

Safety was assessed by monitoring adverse events (AEs) and laboratory parameters.

### Statistical analysis

As this was a pilot study, no formal hypothesis was tested. The sample size was not calculated based on power but rather selected to collect sufficient data to provide a first approximation of the distribution of efficacy and PK results across the large number of formulations that were planned to be studied. The target sample size was 50. All analyses were performed on participants receiving any generic itraconazole and not segregated out by the brand.

Continuous variables were summarized using the number of observations, mean, standard deviation (SD), coefficient of variation (CV), median, and range as appropriate while the categorical values were described using the number of observations and percentages as appropriate. The point estimate and the corresponding 95% confidence intervals (CIs) for the efficacy endpoints were provided. One-Proportion Z-test was used to estimate the 95% CI for the percentage of participants who had a clinical response. The efficacy analyses were performed on the treated population that included participants who had the 7-day treatment regimen, had clinical evaluations at baseline and at Day 7, and completed the study. Missed data at Day 14 follow-up were imputed as “non-responders”. For PK analysis, when an individual concentrate value was below the lower limit of quantification (LLOQ), the concentrate value was assigned a value of 0 for the analysis and presented in tables as below the quantification limit (BQL). In addition, when greater than 50% of the individual concentration values for a given time point were below the LLOQ, the mean, minimum, and median values were reported as BQL, and SD, %CV, and geometric mean were not reported for these analyses. For graphical analysis, plasma concentration values below the LLOQ were displayed as 0 for the linear plots. Logistic regression was used to determine the association of clinical response at Day 7 (dependent variable) with the baseline MIC sensitivity pattern (independent regressor) of causative fungi. SAS version 9.4 was used for calculations.

## Results

### Demographic and baseline characteristics

**Fig 1:**
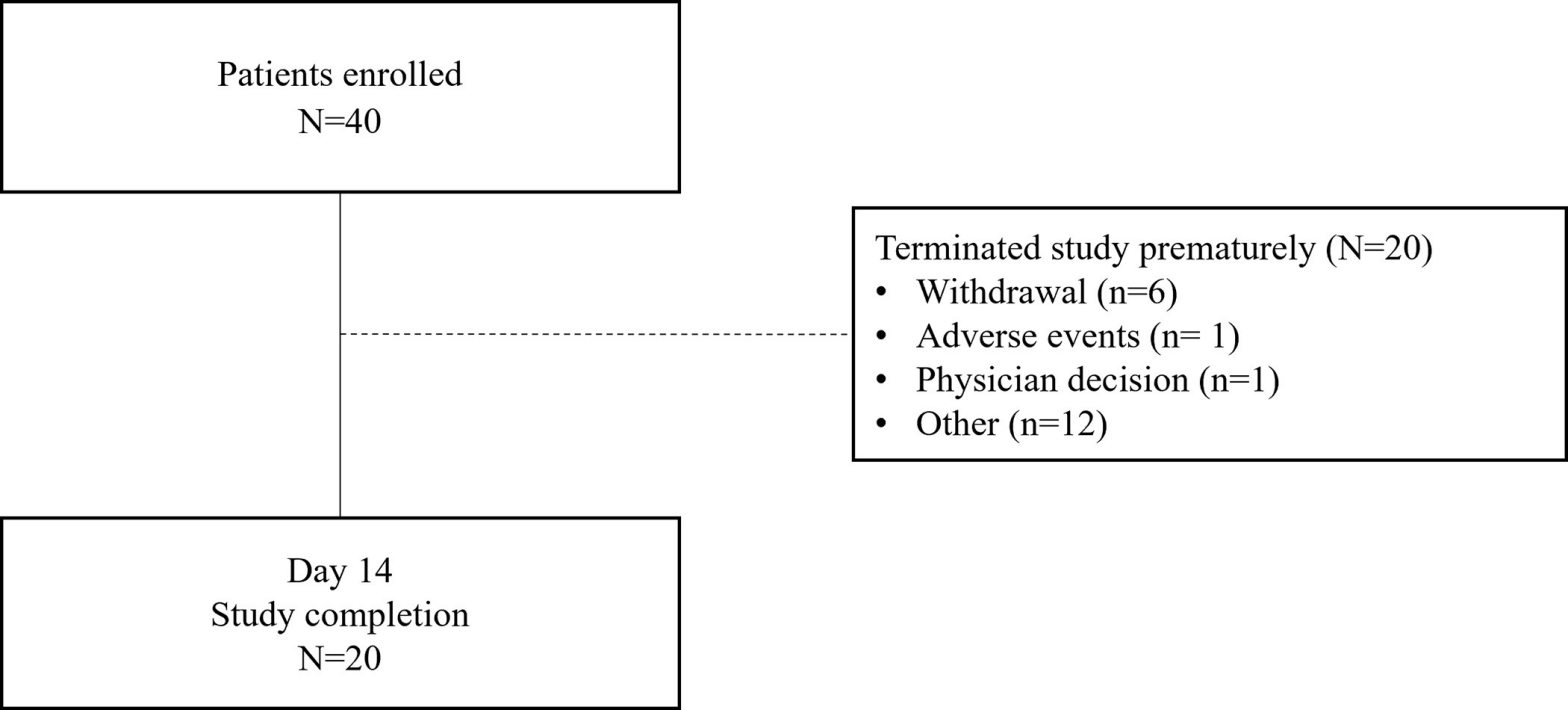
Participant disposition.

The study was conducted from August 2019 to March 2020. Due to COVID-19 related lock-down, the study was prematurely terminated. Forty participants were enrolled of whom 37 received at least one dose of itraconazole, 12 (32%) received 7 days and 25 (67%) received 14 days of itraconazole. A total of 20 participants completed the study (Fig 1). The mean (SD) age of the 40 enrolled participants was 35.5 (12.73) years and 25 [62.5%] were male. The median CET SS total score at baseline was 5.5 (range: 2–10). Of the 11 participants who had taken concomitant medications, the majority (n = 9) had antihistamines for systemic use as a prior medication. The potassium hydroxide (KOH) mount from the skin scrape was negative for 3 and positive for 37 (92.5%) of the 40 participants (Table 1).

**Table 1:** Baseline and disease characteristics of participants.

Parameter	All participants (N = 40)
**Age, years; mean (SD)**	35.5 (12.7)
**Male, n (%)**	25 (62.5%)
**BMI, kg/m** ^**2**^ **; mean (SD)**	25.3 (5.04)
**BSA, m** ^**2**^ **; mean (SD)**	1.733 (0.1565)
**CET SS total score, median (range)**	5.5 (2–10)
**Positive KOH mount, n (%)**	37 (92.5%)

BMI, body mass index; BSA, body surface area; CET SS, Clinical Evaluation Tool Signs and Symptoms; KOH, potassium hydroxide; SD, Standard Deviation.

A total of 28 (75.6%) participants completed the 7-day treatment phase, and only 20 (50%) returned for the Day 14 visit and completed the study as per protocol. The most common reasons for discontinuation were COVID-19-related (n = 6; participants did not continue the site visit due to complete lockdown), personal (n = 6), and patient withdrawal (n = 4). Some participants did not return for either day 7 or day 14 visits. Thus, the number of participants available for clinical response evaluation is less than that on treatment.

### Efficacy

#### Clinical response at day 7

From 28 participants who completed 7 days of treatment and were evaluable for efficacy, 12 (42.9%; 95% CI: 24.53%, 61.19%) had a clinical response as “healed” or “markedly improved” based on the IGE Tool assessment.

#### Clinical response at day 14

Twenty participants completed 14 days of follow-up and were evaluable for clinical response. Eighteen achieved a score of 1 (absence of signs and symptoms) or 2 (≥50% clinical improvement), for clinical response of 90% (95% CI: 76.85%, 100%). When all 8 participants that missed the Day 14 follow-up were imputed as “non-responders”, the estimated proportion of the participants with clinical response was 64.3% (18/28; 95% CI: 46.54%, 82.03%).

### Mycological response

#### Baseline cultures

A total of 28 fungal cultures were obtained at baseline, 24 (85%) were positive. *T*. *mentagrophytes* species was the most common microorganism isolated (91.7%). The culture results were presented in Table 2. All isolates were susceptible to itraconazole.

**Table 2:** Participants with microbial culture results and microbial organism identification (efficacy analysis set).

	n/N (%)^a^
**Screening**	**28**
*Positive*	24/28 (85.7%)
*Trichophyton rubrum*	2/24 (8.3%)
*Trichophyton mentagrophytes* species complex	22/24 (91.7%)
*Negative*	4/28 (14.3%)
**Day 14**	**13**
***Positive***	2/13 (15.4%)
*Trichophyton mentagrophytes* species complex	2/2 (100.0%)
***Negative***	11/13 (84.6%)

^a^Percentages are calculated with respect to the number of participants with available data points at that visit.

#### Cultures at day 14

Of the available culture data for 13 participants, 11 participants had negative cultures, of whom mycological response was achieved by 8 participants (61.5%; 95% CI: 35.09%, 87.98%). Two participants (15.4%) had mycological failure (*T*. *mentagrophytes* species complex isolated at baseline and the Day 14 visit (Table 2), with a clinical response score of 2 (≥50% clinical improvement). All isolates were susceptible to itraconazole.

### Pharmacokinetics

PK analysis included a total of 28 participants who had samples on Day 7, however, PK results on Day 14 were not available for 8 participants as they discontinued the study prior to PK sampling on Day 14. Following oral administration of 200 mg (once daily) itraconazole or reference itraconazole, concentrations of itraconazole and hydroxy-itraconazole were quantifiable in plasma prior to dosing on Day 7 in 23 of the 26 available samples with an LLOQ of 20 ng/mL for both the analytes. Of the 3 participants who had plasma concentrations BQL prior to dosing on Day 7, one participant also had no quantifiable plasma concentrations of itraconazole and hydroxy-itraconazole at 2 hours and 4.5 hours postdose. At Day 7, the mean plasma concentrations for itraconazole and hydroxy-itraconazole in non-responders (participants with a clinical response score of 3 to 5) was comparable to that of responders (participants with a clinical response score of 1 or 2 [healed or markedly improved]; Table 3).

**Table 3:** Plasma concentrations of itraconazole and hydroxy-itraconazole following oral administration of 200 mg itraconazole once daily or reference itraconazole (PK analysis set).

Analyte	Mean (SD) Plasma Concentrations (ng/mL)							
	**Day 7**				**Day 14**			
	**n**	**0h**	**2h**	**4.5h**	**n**	**0h**	**2h**	**4.5h**
**Itraconazole**								
All Participants	28^a^	167 (141)	208 (215)	311 (255)	20	332 (337)	450 (440)	495 (413)
Non-responders	16^b^	170 (152)	229 (230)	304 (288)	2	-	-	-
Responders	12	164 (133)	182 (202)	321 (216)	18	356 (345)	470 (453)	515 (421)
**Hydroxy-itraconazole**								
All Participants	28^a^	377 (267)	388 (301)	441 (287)	20	579 (444)	621 (509)	650 (447)
Non-responders	16^b^	387 (274)	417 (316)	450 (319)	2	-	-	-
Responders	12	364 (271)	353 (290)	429 (250)	18	614 (449)	654 (519)	663 (443)

n = highest observed number of participants across the 3 time points/study day

All Participants = clinical response score 1 to 5, responders = participants with a clinical response score of 1 or 2, non-responders = participants with a clinical response score of 3 to 5

^a^n = 27 for 2h and n = 26 for 0h

^b^n = 15 for 2h and n = 14 for 0h

h, hours; PK, pharmacokinetics; SD, standard deviation

Clinical response at Day 7 was not associated with the highest or lowest plasma concentrations of itraconazole or hydroxy-itraconazole. Of the 3 participants with predose plasma concentrations BQL on Day 7, two participants were classified as responders (clinical response score = 2 [markedly improved]) and 1 participant was classified as a nonresponder (clinical response score = 5 [worse]). All 3 participants had positive fungal cultures for *T*. *mentagrophytes* species complex at screening. On Day 14, 1 of the 3 participants had a positive fungal culture while fungal cultures were not available for the other 2 participants. For most participants (82% [18/22]), the lowest itraconazole drug concentration on Day 7 was greater than the MIC by 1.2 to 19.4 times the MIC at baseline.

### Safety

The safety set included 37 participants who received at least 1 dose of itraconazole. There were no safety concerns with itraconazole. Only one treatment-emergent AE (TEAE) of worsening of *T*. *cruris* infection (moderate intensity) was recorded, leading to the withdrawal of the participant from the study, although, the investigator considered it to be unrelated to the study treatment. No serious AEs or deaths were reported during the study and there were no clinically significant changes in vital signs or laboratory parameters.

## Discussion

This pilot study was planned to determine the clinical response after a treatment course of 7 days of itraconazole in patients suffering from superficial fungal infection in India. However, the study was prematurely terminated due to the COVID-19 pandemic, thus calling for a careful interpretation of the results. Nevertheless, the results provide interesting insights regarding the treatment outcomes and risk factors associated with clinical response in this patient population while they received itraconazole as part of their routine clinical care.

Of the 40 participants enrolled in our study, approximately two-thirds were male, and the mean age was 35.5 years. This is consistent with prior reports showing male preponderance (2–6 times more than females) and relatively high incidence in the age group of 20–40 years in India [2]. We used the CET SS tool, which has been used in the prior itraconazole clinical studies, to assess the presence and severity of signs and symptoms in our study participants and indicated that the disease severity in our study can be considered as mild to moderate (mean CET SS score of 5.5).

A dramatic change in the characteristics of dermatophytosis has been reported in India in recent years and *Trichophyton* species has emerged as a predominant organism with *T*. *rubrum*, *T*. *mentagrophytes* and *T*. *verrucosum* are the most commonly identified [32]. The culture results in our study supported these findings as *T*. *mentagrophytes* was the most common species (~92%) identified followed by *T*. *rubrum* (~8%). The MIC for itraconazole was within the susceptibility range (0.015 to 0.25 mcg/mL) in all isolates both at baseline and after treatment. At Day 7 more than 40% of the participants achieved clinical response (healed or markedly improved). There was approximately a 2-fold improvement in the clinical response at Day 14 compared with Day 7 based on the completer analysis. This estimated ratio became 1.5 when participants who missed the Day 14 follow-up were categorized as “non-responders”. The observed results are similar to those of the pivotal studies of itraconazole where >80% superficial dermatophytosis healed with the use of 100 mg oral itraconazole for 7 days [33].

The PK properties of itraconazole should also be evaluated while considering the dose and duration of treatment [8]. Itraconazole has non-linear pharmacokinetics which contributes to its unpredictable absorption patterns. The observed absolute oral bioavailability of itraconazole is about 55%; however, gastric conditions (fed vs fasting) and other concomitant medications affecting gastric acid secretion results in inter-patient variability [9, 34]. On the other hand, itraconazole is rapidly absorbed after oral administration reaching peak plasma concentrations within 2–5 hours following an oral capsule dose and steady-state concentrations are reached by 14–15 days following daily dosing of up to 400 mg [35].

Interestingly, in our study, there was a lack of correlation between PK results and the clinical outcomes observed, along with an absence of itraconazole resistance in the causative organisms. The clinical response at Day 7 was neither associated with the highest or lowest observed plasma concentrations of itraconazole and hydroxy-itraconazole, nor with the MIC of the causative fungi. Thus, the treatment failure could not be attributed to either a lack of drug exposure or elevated MIC values for *T*. *mentagrophytes* species complex. This might be due to the fact that itraconazole accumulates at the infection site, making consistently high plasma concentrations unnecessary for clinical response [29]. Owing to its high lipophilicity itraconazole exhibits up to 4-times higher concentrations in skin tissue compared to the plasma concentration and although the uptake is slower and dependent on the skin areas, peak concentrations are observed at 7–14 days in the stratum corneum and persist for up to 2–4 weeks after the discontinuation of therapy [36–38].

As per the recommendations of the Expert Consensus on the Management of Dermatophytosis in India (ECTODERM India), itraconazole is the preferred systemic antifungal therapy. However, in view of the perceived changes in both prevalence and clinical characteristics of dermatomycosis in India (i.e., more severe and recalcitrant), the ECTODERM expert panel recommended itraconazole doses are 100–200 mg once a day for 2–4 weeks in treating naïve cases, and 200–400 mg once a day prolonged periods (>4 weeks) in case of recalcitrant cases [2].

Anecdotal reports of increased treatment failures with the use of itraconazole in India should be interpreted with caution. Although this is a small study, our findings suggest that resistance to itraconazole or PK factors may not be the cause of those reported findings. There are however other factors that cannot be excluded. One of them is the severity of the disease—based on our clinical evaluation the cases included in this study were mild. It is possible that in more severe cases the response to itraconazole would be different. Another factor is adherence to treatment. It is well known that patients included in clinical trials have better adherence to treatment. Although this study tried to assess these patients in their regular practice, we included evaluations that were not part of their regular clinical care and that could have influenced their adherence to therapy and therefore the observed clinical outcomes. There have been other efforts to understand the epidemic of dermatophytosis in India with similar results [6, 32, 39, 40].

The conduct of the study was difficult from many perspectives; the COVID-19 pandemic affected patient recruitment and follow-up and consequently, the study understandably suffered from a significant drop-out rate. Though the small sample size and high discontinuation rate are important limitations, the study reconfirmed the clinical efficacy and microbial susceptibility to itraconazole for the fungi causing dermatophytosis in India.

## Supporting information

S1 Checklist (DOCX)

S1 File (PDF)

S2 File (PDF)

S3 File (PDF)

S4 File (DOCX)
