# Supplementary material for: No evidence of resistance to itraconazole in a prospective real-world trial of dermatomycosis in India
Source: PLoS One. 2023 Feb 14;18(2):e0281514. doi: 10.1371/journal.pone.0281514 (PMC9928099; doi:10.1371/journal.pone.0281514)
Supplement: S2 File — (PDF) [file pone.0281514.s003.pdf]

Janssen Research & Development *

Clinical Protocol

Itraconazole in the Management of Superficial Fungal Infections in India: A Pilot Study.

Protocol R051211FUN4058; Phase 4
Amendment 1
JNJ-16269994 (itraconazole)

*Janssen Research & Development is a global organization that operates through different legal entities in various countries. Therefore, the legal entity acting as the sponsor for Janssen Research & Development studies may vary, such as, but not limited to Janssen Biotech, Inc.; Janssen Products, LP; Janssen Biologics, BV; Janssen-Cilag International NV; Janssen, Inc; Janssen Pharmaceutica NV; Janssen Sciences Ireland UC; or Janssen Research & Development, LLC. The term "sponsor" is used throughout the protocol to represent these various legal entities; the sponsor is identified on the Contact Information page that accompanies the protocol.

Status: Approved
Date: 8 February 2019
Prepared by: Janssen Research & Development, LLC
EDMS number: EDMS-ERI-160717658, 2.0

GCP Compliance: This study will be conducted in compliance with Good Clinical Practice, and applicable regulatory requirements.

[redacted]

PROTOCOL AMENDMENT SUMMARY OF CHANGES TABLE

DOCUMENT HISTORY	
**Document**	**Date**
Amendment 1	08-Feb-2019
Original Protocol	28-Jun-2018

Amendment 1 (08 February 2019)

Overall Rationale for the Amendment: Secondary study objective/endpoint that compares subjects treated with generic itraconazole versus SPORANOX was deleted. Reference to the word "SPORANOX" was also deleted and replaced with "reference itraconazole". This was done at the request of the Health Authority in India, the Directorate of General of Health Services, due to their claims that the main scope of this study should not be a comparison between various brands of itraconazole.

Section Number and Name	Description of Change	Brief Rationale
Synopsis, Objectives, Statistical Methods;<br>3 Objectives;<br>9.4.1 Efficacy Analyses	Secondary objective/endpoint that estimates the proportion of participants that received generic itraconazole versus branded SPORANOX was deleted.	At the request of the Health Authority in India, the Directorate General of Health Services, the aforementioned secondary objective/endpoint was deleted because the main scope of this study should not be a comparison between brands of itraconazole.
Synopsis Overview, Objectives, Design, Number of Participants, SoA, Pharmacokinetic and Sensitivity Evaluations,<br>1.2 Schema;<br>2.1 Study Rationale;<br>2.2 Background;<br>2.3 Benefit/Risk Assessment;<br>3 Objectives;<br>4.1 Design;<br>4.2 Scientific Rationale for Study Design;<br>4.2.1 Study-Specific Ethical Design Considerations;<br>4.3 Justification for Dose;<br>5 Study Population;<br>5.1 Inclusion Criteria;<br>6.1 Study Interventions Administered;	Reference to "generic itraconazole" and "SPORANOX" was deleted and replaced with "itraconazole" or "reference itraconazole".	At the request of the Health Authority in India, the Directorate General of Health Services, the main scope of this study should not be a comparison between brands of itraconazole.

**Section Number and Name**	**Description of Change**	**Brief Rationale**
6.4 Study Intervention Compliance;<br>9.2 Sample Size Determination;<br>9.4.1 Efficacy Analyses;<br>9.4.3 Pharmacokinetic Analyses		
Synopsis, Number of Participants, Statistical Methods;<br>2.1 Study Rationale;<br>4.1 Overall Design;<br>9.2 Sample Size Determination	Study population was increased from 26 to 50 subjects.	The Health Authority in India, the Directorate General of Health Services, requested a higher sample size for this study due to concerns that the actual situation of the disease in the country would not allow us to draw any conclusions with the proposed sample size.
Synopsis, Statistical Methods<br><br>9.1 Statistical Hypotheses<br><br>9.4.2 Safety Analyses;<br>9.4.3 Pharmacokinetic Analyses	Rationale for sample size selection was changed to explain methodology for conducting efficacy and PK analyses across a large number of formulations.<br><br>Confidence interval for the difference in response rates between generic itraconazole and SPORANOX will no longer be conducted.<br><br>Summaries by treatment groups were deleted.	No analysis will be conducted on the difference between generic itraconazole and SPORANOX, but rather across different itraconazole products, as the main scope of this study should not be a comparison between various brands of itraconazole.
4.2 Scientific Rationale	The overall distribution of plasma concentration of itraconazole and hydroxy-itraconazole will be determined across different products rather than in generic itraconazole versus SPORANOX.	The main scope of this study should not be a comparison between various brands of itraconazole.
2.3 Benefit/Risk Assessment	Footnote added to explain information for SPORANOX as the sponsor's itraconazole brand.	Since the benefit/risk information specifically pertains to SPORANOX, the sponsor's itraconazole brand, reference to "SPORANOX" could not be deleted as per the HA's request. Instead, a footnote explanation was added.
Throughout the protocol	Minor grammatical, formatting, redundancies or spelling changes were made.	Minor errors were noted.

TABLE OF CONTENTS

PROTOCOL AMENDMENT SUMMARY OF CHANGES TABLE 2

TABLE OF CONTENTS 4

LIST OF IN-TEXT TABLES AND FIGURES 6

1. PROTOCOL SUMMARY 7
1.1. Synopsis 7
1.2. Schema 13
1.3. Schedule of Activities (SoA) 14

2. INTRODUCTION 16
2.1. Study Rationale 16
2.2. Background 17
2.3. Benefit/Risk Assessment 20

3. OBJECTIVES AND ENDPOINTS 22

4. STUDY DESIGN 22
4.1. Overall Design 22
4.2. Scientific Rationale for Study Design 24
4.2.1. Study-Specific Ethical Design Considerations 25
4.3. Justification for Dose 25
4.4. End of Study Definition 25

5. STUDY POPULATION 25
5.1. Inclusion Criteria 26
5.2. Exclusion Criteria 27
5.3. Lifestyle Considerations 28
5.4. Screen Failures 28

6. STUDY INTERVENTION 29
6.1. Study Interventions Administered 29
6.2. Preparation/Handling/Storage/Accountability 29
6.3. Measures to Minimize Bias: Randomization and Blinding 29
6.4. Study Intervention Compliance 29
6.5. Concomitant Therapy 29
6.5.1. Prohibited Medication 30
6.6. Dose Modification 30
6.7. Intervention After the End of the Study 30

7. DISCONTINUATION OF STUDY INTERVENTION AND PARTICIPANT DISCONTINUATION/WITHDRAWAL 30
7.1. Discontinuation of Study Intervention 30
7.2. Participant Discontinuation/Withdrawal From the Study 31
7.3. Lost to Follow-up 31

8. STUDY ASSESSMENTS AND PROCEDURES 32
8.1. Efficacy Assessments 33
8.2. Safety Assessments 33
8.2.1. Clinical Safety Laboratory Assessments 33
8.3. Adverse Events and Serious Adverse Events 34
8.3.1. Time Period and Frequency for Collecting Adverse Event and Serious Adverse Event Information 34
8.3.2. Method of Detecting Adverse Events and Serious Adverse Events 35
8.3.3. Follow-up of Adverse Events and Serious Adverse Events 35
8.3.4. Regulatory Reporting Requirements for Serious Adverse Events 35

8.3.5. Pregnancy 35
8.4. Treatment of Overdose 35
8.5. Pharmacokinetics 36
8.5.1. Sample Collection and Handling 36
8.5.2. Analytical Procedures 36
8.7. Genetics 36
8.8. Biomarkers 36

9. STATISTICAL CONSIDERATIONS 36
9.1. Statistical Hypotheses 37
9.2. Sample Size Determination 37
9.3. Populations for Analyses 37
9.4. Statistical Analyses 38
9.4.1. Efficacy Analyses 38
9.4.2. Safety Analyses 39
9.4.3. Pharmacokinetic Analyses 39
9.5. Interim Analysis 39

10. SUPPORTING DOCUMENTATION AND OPERATIONAL CONSIDERATIONS 40
10.1. Appendix 1: Abbreviations and Trademarks 41
10.2. Appendix 2: Clinical Assessment Tool 42
10.3. Appendix 3: Contraceptive and Barrier Guidance and Collection of Pregnancy Information 43
10.4. Appendix 4: Regulatory, Ethical, and Study Oversight Considerations 46
REGULATORY AND ETHICAL CONSIDERATIONS 46
FINANCIAL DISCLOSURE 49
INFORMED CONSENT PROCESS 49
DATA PROTECTION 50
LONG-TERM RETENTION OF SAMPLES FOR ADDITIONAL FUTURE RESEARCH 50
COMMITTEES STRUCTURE 50
PUBLICATION POLICY/DISSEMINATION OF CLINICAL STUDY DATA 50
DATA QUALITY ASSURANCE 52
CASE REPORT FORM COMPLETION 52
SOURCE DOCUMENTS 53
MONITORING 53
ON-SITE AUDITS 54
RECORD RETENTION 54
STUDY AND SITE CLOSURE 55
10.5. Appendix 5: Adverse Events: Definitions and Procedures for Recording, Evaluating, Follow-up, and Reporting 56
ADVERSE EVENT DEFINITIONS AND CLASSIFICATIONS 56
ATTRIBUTION DEFINITIONS 57
SEVERITY CRITERIA 57
SPECIAL REPORTING SITUATIONS 58
PROCEDURES 58
CONTACTING SPONSOR REGARDING SAFETY 59
PRODUCT QUALITY COMPLAINT HANDLING 60
10.6. Appendix 6: Clinical Laboratory Tests 61
10.7. Appendix 7: Protocol Amendment History 62

11. REFERENCES 63

INVESTIGATOR AGREEMENT 65

LIST OF IN-TEXT TABLES AND FIGURES

TABLES

Table 1: Clinical Evaluation Tool: Signs and Symptoms 42
Table 2: Investigator Global Evaluation Tool: Clinical Response 42
Table 3: Protocol-Required Safety Laboratory Assessments 61
Table 4: Protocol-Required Laboratory Assessments 61

FIGURES

Figure 1: Schematic Overview of the Study 13

1. PROTOCOL SUMMARY

1.1. Synopsis

Itraconazole in the Management of Superficial Fungal Infections in India: A Pilot Study.

Over the last 3–4 years, practitioners in India have observed an increased frequency of dermatomycosis and treatment failure. Due to limited alternatives, oral itraconazole has become treatment of choice in the recent years in the country. However, there are now unconfirmed concerns of emergence of failures with the use of itraconazole for the treatment of dermatomycosis. There is a need to evaluate the magnitude of the problem in India and the risk factors associated with poor clinical response to the use of itraconazole in patients with dermatomycosis. This pilot study will help understand the feasibility and challenges associated with conducting the real-world study in India for dermatomycosis and to make modifications in the design of the full study as appropriate.

This pilot study will be performed as a real-world clinical evaluation of participants that receive oral itraconazole, including the sponsor's itraconazole brand which will be noted as "reference itraconazole", as part of their clinical care for the treatment of *Tinea cruris* or *Tinea corporis*. The systematic evaluation of clinical response, cultures of causative organism from the lesions and determination of plasma concentrations of itraconazole and hydroxy-itraconazole will be the study interventions evaluated in this study.

OBJECTIVES AND ENDPOINTS

Objectives	Endpoints
**Primary**	
• Estimate the proportion of participants prescribed itraconazole for *T. cruris* or *T. corporis* who have clinical response after 7 days of treatment	• Proportion of participants with clinical response (defined as "healed" or "markedly improved" based on the Investigator Global Evaluation Tool of clinical improvement [see Appendix 10.2]) at the end of 7 days of treatment
**Secondary**	
• Estimate the proportion of participants prescribed itraconazole for *T. cruris* or *T. corporis* who have mycological response after 14 days of follow-up	• Mycological cure defined as both culture and microscopy negative
• Association of the clinical response at Day 7 with plasma drug concentrations of itraconazole and hydroxy-itraconazole	• Plasma concentrations of itraconazole and hydroxy-itraconazole during therapy
• Association of the clinical response at Day 7 with the baseline sensitivity pattern of causative fungi	• Baseline minimum inhibitory concentration (MIC)
• Estimate proportion of participants with clinical response after 14 days of follow-up. Estimate the extent to which clinically improved at Day 7 predicts clinically improved at Day 14	• Proportion of participants with clinical response (defined as "healed" or "markedly improved" based on the Investigator Global Evaluation Tool of clinical improvement [see Appendix 10.2]) at the end of 14 days of follow-up

Objectives	Endpoints
**Tertiary/Exploratory**	
• Evaluate the proportion of participants that come for follow-up after 7 days of treatment	• Proportion of participants coming for evaluation after 7 days of treatment

OVERALL DESIGN

This is a real-world, prospective, non-randomized, open-label, multicenter, interventional, longitudinal pilot study to evaluate clinical outcomes and risk factors associated with clinical response in adult participants from India with *T. cruris* or *T. corporis* treated with itraconazole as part their clinical care. The interventions in this study will be the systematic evaluation of clinical response, cultures of causative organism from the lesions and determination of plasma concentrations of itraconazole and hydroxy-itraconazole.

The study will consist of 3 phases: a screening phase; a 7-day open-label, treatment phase; a 7-day observation phase; and end of study (EOS) visit at Day 14. The duration of individual participation will be approximately 15 days.

NUMBER OF PARTICIPANTS

Fifty adult men and women (18 to 60 years of age) with *T. cruris* or *T. corporis* infection that have been prescribed itraconazole 200 mg daily will be recruited from clinical practices of dermatologists in India. The study will be conducted at 4 to 5 large clinics of dermatologists across different regions of India and will include sites that have their own pharmacies that dispense itraconazole and reference itraconazole. A minimum of 6 participants treated with reference itraconazole will be included or the mitigation plan of study extension will be implemented.

INTERVENTION GROUPS AND DURATION

At baseline, all participants with *T. cruris* or *T. corporis* that have been prescribed itraconazole 200 mg daily by their treating physician as part of their clinical care and meet eligibility criteria will undergo a clinical evaluation of signs and symptoms using an Investigator Global Evaluation Tool and skin scraping collection for potassium hydroxide (KOH) mount and culture and drug sensitivity. The brand and strength (100 mg or 200 mg) of itraconazole will be determined by the treating physician and local pharmacies where itraconazole is dispensed. Participants will receive their regular clinical care at the discretion of their treating physician and will enter the open-label, treatment phase and take 200 mg of itraconazole daily with a full meal on Day 1 through Day 7. Clinical response using the Investigator Global Evaluation Tool (see Appendix 10.2) will be done at Day 7 (first assessment visit) and on Day 14 (second assessment visit). At first assessment visit on Day 7, participants will undergo collection of 3 blood samples to determine plasma concentration of itraconazole and its main metabolite, hydroxy-itraconazole. At the EOS visit on Day 14, skin scraping will be collected for KOH mount and culture. Treatment duration will be determined at the discretion of the physician. Only those participants that continue receiving the study drug at the second assessment visit on Day 14 will undergo collection of 3 blood samples to determine plasma concentration of itraconazole and hydroxy-itraconazole.

Schedule of Activities (SoA)

Phase	Screening	Open-Label: Treatment[a]						Open-Label: Observation					End of Study Visit
Day (D)	-3 to 0	1 to 6	7				8 to 13	14				14
Time			Pre-dose	0H	2H	4.5H		Pre-dose	0H	2H	4.5H	
**Study Procedure**												
Informed consent Form (ICF)[b]	X											
Demographics	X											
Review medical history requirements	X											
Inclusion/exclusion criteria	X											
Clinical evaluation of signs and symptoms[c]	X			X								X
Pregnancy confirmation test (Serum β-hCG)[d]	X											
Itraconazole intake by participants		X		X[e]								
Adverse events[f]				X								X
Treatment compliance via capsule counting				X								X[g]
Concomitant therapy[h]	X			X								X[g]
**Specific procedures**												
Skin scraping collection for culture and sensitivity to itraconazole	X											X
Skin scraping collection for KOH mount	X											X
Sample collection for plasma level of itraconazole			X[i]		X	X		X[i,g]		X[g]	X[g]	

a Treatment duration determined at discretion of treating physician as standard practice.
b Must be signed before first study-related activity.
c Assessment of clinical response will be done using an Investigator Global Evaluation Tool based on score of signs and symptoms at baseline and follow-up visits.
d Mandatory for female in reproductive age group.
e To be taken in clinic after collection of first blood sample.
f Adverse events can be reported at any time during the study. It will be actively asked during the follow-up visits.
g Only in participants that continue receiving itraconazole at the discretion of the treating physician as a standard of practice.

h All previous therapies taken 14 days before the first dose of itraconazole must be recorded at screening. Concomitant therapies should be recorded beyond 14 days if they are associated with serious adverse events. Capture of antipruritic drugs are mandatory.

i To be collected 24 hrs ± 2 hrs after previous dose (D6, D13).

Key: β-hCG=beta human chorionic gonadotropin; D=Day; H=hours; ICF=informed consent form; KOH=potassium hydroxide; SoA=Schedule of Activities.

EFFICACY EVALUATIONS

Clinical efficacy will be evaluated with the Clinical Assessment Tools. The investigator will use the Clinical Evaluation Tool to assess the severity of signs and symptoms with a total score ranging from 0 to 18 at each visit (baseline, at Day 7, and at Day 14). The total scores at Day 7 and at Day 14 compared with baseline scores will be used to define the percentage of clinical improvement. The percentage of clinical improvement will be used to classify the clinical efficacy using the Investigator Global Evaluation Tool (Table 2 of Appendix 10.2). A score from 1 to 5 will be assigned at Day 7 and at Day 14 based on the percentage of clinical improvement (Table 2). Clinical Response will be defined as having scores 1 or 2 ("healed" or "markedly improved").

Skin scraping will be collected at the EOS visit on Day 14 for KOH mount and culture to evaluate mycological cure. A participant will be defined as mycologically cured upon negative culture and microscopy result at the end of follow-up.

PHARMACOKINETIC AND SENSITIVITY EVALUATIONS

Venous blood samples will be collected at timepoints as specified in the Schedule of Activities (SoA) to measure plasma concentrations of itraconazole and hydroxy-itraconazole.

Skin scrapings collection for KOH mount and culture and drug sensitivity at baseline and Day 14 will be conducted to identify fungal pathogen and its sensitivity to itraconazole. Baseline minimum inhibitory concentration (MIC) for itraconazole will be correlated to clinical outcome.

SAFETY EVALUATIONS

Safety evaluations will be conducted at discretion of the treating physician as part of their standard practice. Adverse events will be reported and followed by the investigator and recorded on the Case Report Forms (CRFs).

A serum pregnancy test will be performed at screening.

STATISTICAL METHODS

No formal hypothesis testing will be conducted for this pilot study. Data will be summarized using descriptive statistics. Continuous variables will be summarized using the number of observations, mean, standard deviation, coefficient of variation, median, and range as appropriate. Categorical values will be summarized using the number of observations and percentages as appropriate. Summary statistics will be provided for each treatment group.

The sample size was not calculated based on power, rather it was selected in order to collect sufficient data to give a first approximation of the distribution of efficacy and pharmacokinetic (PK) results across the large number of formulations that will ultimately be studied. Fifty subjects will be enrolled.

Primary Endpoint

The primary efficacy endpoint is defined as the estimated proportion of participants who have clinical response ("healed" or "markedly improved" of the Investigator Global Evaluation Tool of clinical improvement) after 7 days of treatment. The primary analysis will be performed on all participants that have clinical evaluations at baseline and Day 7 after 7 days of treatment. A secondary analysis will be performed on the PK population, defined as all treated participants who have at least one set of blood samples drawn at Day 7 to evaluate plasma drug concentrations of itraconazole and its main metabolite, hydroxy-itraconazole.

Major Secondary Endpoints

Major secondary endpoints include:

- Estimate the proportion of participants prescribed itraconazole who have mycological response after 14 days of follow-up.
- Association of the clinical outcome with plasma concentrations of itraconazole and hydroxy-itraconazole.
- Association of the clinical outcome with baseline MIC of causative fungi.
- Estimation of the proportion of participants with clinical response after 14 days of follow-up. Estimate the extent to which clinically improved at Day 7 predicts clinically improved at Day 14.

Safety Analysis

Safety analysis will include summaries of adverse events (AEs). These summaries will be based on participants who received at least 1 dose of study drug.

1.2. Schema

Figure 1: Schematic Overview of the Study

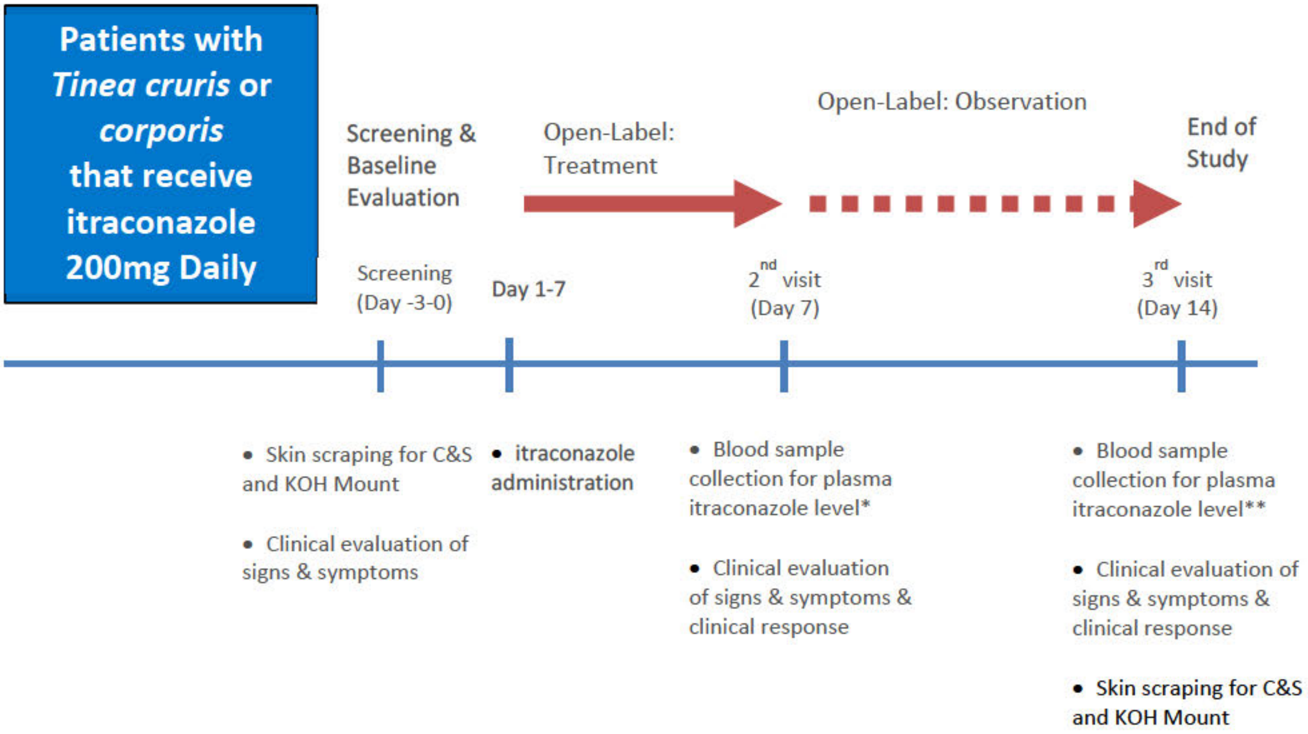

*Blood Samples Collection: 24±2 hours after previous dose (Day 6, Day 13) and 2 and 4.5 hours ±10 minutes after the last dose (Day 7, Day 14).

**Only in participants that continue receiving itraconazole at the discretion of the treating physician as standard practice.

Key: C&S=culture and itraconazole sensitivity; KOH=potassium hydroxide.

1.3. Schedule of Activities (SoA)

Phase	Screening	Open-Label: Treatment[a]					Open-Label: Observation					End of Study Visit
Day (D)	-3 to 0	1 to 6	7				8 to 13	14				14
Time			Pre-dose	0H	2H	4.5H		Pre-dose	0H	2H	4.5H	
**Study Procedure**												
Informed consent Form (ICF)[b]	X											
Demographics	X											
Review medical history requirements	X											
Inclusion/exclusion criteria	X											
Clinical evaluation of signs and symptoms[c]	X		X									X
Pregnancy confirmation test (Serum β-hCG)[d]	X											
Itraconazole intake by participants		X		X[e]								
Adverse events[f]			X									X
Treatment compliance via capsule counting			X									X[g]
Concomitant therapy[h]	X		X									X[g]
**Specific procedures**												
Skin scraping collection for culture and sensitivity to itraconazole	X											X
Skin scraping collection for KOH mount	X											X
Sample collection for plasma level of itraconazole			X[i]		X	X		X[i,g]		X[g]	X[g]	

a Treatment duration determined at discretion of treating physician as standard practice.
b Must be signed before first study-related activity.
c Assessment of clinical response will be done using an Investigator Global Evaluation Tool based on score of signs and symptoms at baseline and follow-up visits.
d Mandatory for female in reproductive age group.
e To be taken in clinic after collection of first blood sample.
f Adverse events can be reported at any time during the study. It will be actively asked during the follow-up visits.
g Only in participants that continue receiving itraconazole at the discretion of the treating physician as a standard of practice.

h All previous therapies taken 14 days before the first dose of itraconazole must be recorded at screening. Concomitant therapies should be recorded beyond 14 days if they are associated with serious adverse events. Capture of antipruritic drugs are mandatory.

i To be collected 24 hrs ± 2 hrs after previous dose (D6, D13).

Key: β-hCG=beta human chorionic gonadotropin; D=Day; H=hours; ICF=informed consent form; KOH=potassium hydroxide; SoA=Schedule of Activities.

2. INTRODUCTION

Itraconazole is an oral triazole derivative with a broad spectrum of activity. In vitro studies have demonstrated that itraconazole impairs the synthesis of ergosterol in fungal cells. Ergosterol is a vital cell membrane component in fungi. Impairment of its synthesis ultimately results in an antifungal effect. The safety profile of itraconazole has been studied extensively.

Itraconazole is currently approved globally, including United States, European Union, India, China and Canada for a wide range of fungal infections which include treatment of dermatomycosis, onychomycosis, vaginal candidiasis and pityriasis versicolor. It has also been shown to be effective in systemic mycoses. For a comprehensive list of indications see the latest version of Summary of Product Characteristics (SmPC).[20]

Superficial fungal skin infections include infections of the skin and the scalp (dermatomycosis). Dermatomycosis can be caused by a variety of fungal organisms amongst which dermatophytes are by far the most frequent.[5,10,11,14,17,18,22] Dermatomycosis are commonly classified according to the initial site of infection. *Tinea corporis* comprises the dermatophyte infections of the glabrous skin, with exclusion of certain locations, such as groin, palms and soles. *Tinea cruris* is a dermatophyte infection affecting the groin, including the genitalia, pubic area, perinea and perianal skin, and is seen more often in males.[12,17]

The diagnosis of most dermatomycosis is based on clinical features and mycological investigation. Oral therapy is usually preferred for the more chronic, extensive, and severe forms of dermatomycosis. For *T. cruris* or *T. corporis*, the approved dose of itraconazole is at is 100 mg daily for 15 days or 200 mg daily for 7 days.

For the most comprehensive nonclinical and clinical information regarding itraconazole, refer to the latest version of the Investigator's Brochure (IB)[8] for itraconazole and the SmPC[20].

The term "sponsor" used throughout this document refers to the entities listed in the Contact Information page(s), which will be provided as a separate document.

2.1. Study Rationale

Superficial cutaneous fungal infections or dermatomycosis of the skin, hair, and nail are among the most common infective dermatoses seen in dermatology outpatient clinics in India. Today, according to statements by practitioners, India is facing an increase of chronic and recurrent dermatomycosis. Over the last 3–4 years, the frequency of such cases is believed to have increased substantially[7]. Chronic and recurrent dermatophyte infections cause major distress to the patients socially, emotionally, and financially.[7,24,25]

Many antifungal agents indicated for the treatment of dermatomycosis have been introduced in India in the past. However, during recent years, an increased frequency of treatment failure has been observed. This increase of treatment failure has been attributed to multiple factors including the misuse of antifungal agents, the widespread use of high potency steroids in topical formulations and potential low quality of generics.[25] It has also been suggested that there is emergence of

antifungal resistance that has precluded the use of terbinafine, until recently considered as preferred option for the treatment of dermatomycosis in India.[13] Due to limited alternatives, oral itraconazole has become treatment of choice in the recent years in the country.[7] However, there are now unconfirmed reports of emergence of failures with the use of itraconazole for the treatment of dermatomycosis. Treatment failures could arise from reduced or variable plasma levels of itraconazole and/or hydroxy-itraconazole resulting from inter-individual variability in drug absorption and or differences or inconsistencies in formulations used in treatment.

There is a need to evaluate the magnitude of the problem in India and the risk factors associated with poor clinical response to the use of itraconazole in patients with dermatomycosis. While the literature and reviews from experts in India suggest various reasons for this scenario, there is no strong clinical evidence that supports any of these hypothesis.[15] Knowing the magnitude of the problem and the risk factors associated with it, constitute an important step to implement informed measures to improve the prognosis in this patient population and to prevent or slow dissemination of antifungal resistance.

Therefore, this study is designed to perform real-world clinical investigation that evaluates treatment outcomes and risk factors associated with clinical response in patients with dermatomycosis treated with itraconazole in India. The prospective observational study is planned to be conducted in two phases – pilot and main. The pilot is proposed with the objective to understand the feasibility and challenges associated with conducting the real-world study in India for dermatomycosis and to make modifications in the design of the full study as appropriate. This pilot study will obtain information on 50 participants that will be similar to the information that will be gathered in the main study. The objective of the main study is to estimate the proportion of participants with *T. cruris* or *T. corporis* who will have clinical response after 7 days of treatment with oral itraconazole (and as per the standard of practice in India). The associated baseline resistance will also be determined for these participants from plasma concentration of itraconazole. To meet these objectives, participants will be recruited from 4 to 5 large clinics of dermatologists across different regions of India, and will include sites that have their own pharmacies that dispense itraconazole including the sponsor's itraconazole brand which will be noted as "reference itraconazole" from hence forth. After the inclusion of 12 participants, the number of participants taking reference itraconazole will be evaluated to ensure that a minimum 6 participants will be included. Unless there are changes between the pilot study and the main study that would make it infeasible to do so, the data in this pilot study will be included in the analysis of the main study.

2.2. Background

Itraconazole is active against infections caused by dermatophytes (*Trichophyton* species [spp.], *Microsporum* spp., *Epidermophyton floccosum*), yeasts (*Candida* spp., including *C. albicans*, *C. glabrata* and *C. krusei*, *Cryptococcus neoformans* and *Malassezia* spp.), *Aspergillus* spp., *Histoplasma* spp., *Paracoccidioidis brasiliensis*, *Blastomyces dermatitidis*, *Fonsecaea* spp., *Dermatiaceous hyphomycetes* and many other fungi. The high activity of itraconazole is ascribed to its prominent affinity to fungal cytochrome P450, which is involved in the biosynthesis of ergosterol from lanosterol. Ergosterol is a vital cell membrane component in fungi and its specific inhibition by itraconazole results in drastic inhibition of fungal growth. In vitro, itraconazole

inhibits most isolates of pathogenic yeast species, dermatophytes and *Aspergillus* spp. at concentrations below 1 µg/mL. In guinea pigs, experimentally infected with *Microsporum canis* or *Trichophyton mentagrophytes*, the dermatophytosis was cured or substantially improved in the majority of animals at 2.5 mg/kg/day and cured in all animals at doses at or above the level of 5 mg/kg/day.[9]

For the safety evaluation of repeated oral use of itraconazole, rats were dosed daily for 3, 6 and 12 months and dogs for 3 and 12 months. No toxic effects were observed in rats dosed at 2.5 and 10 mg/kg, except for a slight increase in serum cholesterol and phospholipids, which was observed at all tested doses. The no-toxic-effect dose in dogs was 5 mg/kg. At higher doses, the main targets for toxicity were the adrenal cortex, the ovaries (rats), the mononuclear phagocyte system, the liver, the bone (rats dosed for ≥6 months) and the kidneys (rats dosed for ≥6 months). The clinical relevance for each of these targets was assessed to be within acceptable limits in patients.[9]

No primary carcinogenic effects were observed in mice up to 80 mg/kg (23 months oral treatment) and in rats up to 20 mg/kg (24 months oral treatment).[9]

Itraconazole was not primary antifertile. It did not produce primary adverse effects on reproduction in mice, rats or rabbits. However, at maternally toxic doses, it has, as is the case for other systemically active antifungal azoles, embryotoxic and teratogenic properties in rodents. These properties are, at least partially, related to the adrenocortical effects. No mutagenic effects were found in any of the mutagenicity studies.[9]

Itraconazole is rapidly absorbed after oral administration in humans. Peak plasma concentrations of the unchanged drug are reached within 2 to 5 hours following an oral capsule dose. The observed absolute oral bioavailability of itraconazole is about 55%. Oral bioavailability is maximal when the capsules are taken immediately after a full meal. Itraconazole exposure is lower with the capsule formulation than with the oral solution when the same dose of drug is given. Most of the itraconazole in plasma is bound to protein (99.8%), with albumin being the main binding component (99.6% for the hydroxy-metabolite). It has also a marked affinity for lipids. Only 0.2% of the itraconazole in plasma is present as free drug. Itraconazole is distributed in a large apparent volume in the body (>700 L), suggesting extensive distribution into tissues. Itraconazole is extensively metabolized by the liver into a large number of metabolites. In vitro studies have shown that CYP3A4 is the major enzyme involved in the metabolism of itraconazole. The main metabolite is hydroxy-itraconazole, which has in vitro antifungal activity comparable to itraconazole; trough plasma concentrations of this metabolite are about twice those of itraconazole. Itraconazole is excreted mainly as inactive metabolites in urine (35%) and feces (54%) within one week of an oral solution dose. Renal excretion of itraconazole and the active metabolite hydroxy-itraconazole account for less than 1% of an intravenous dose. Based on an oral radiolabeled dose, fecal excretion of unchanged drug ranges from 3% to 18% of the dose.[6]

As a consequence of non-linear pharmacokinetics, itraconazole accumulates in plasma during multiple dosing. Steady-state concentrations are generally reached within about 15 days, with Cmax values of 0.5 µg/mL, 1.1 µg/mL and 2.0 µg/mL after oral administration of 100 mg once daily, 200 mg once daily and 200 mg twice daily, respectively. The terminal half-life of

itraconazole generally ranges from 16 to 28 hours after single dose and increases to 34 to 42 hours with repeated dosing. Once treatment is stopped, itraconazole plasma concentrations decrease to an almost undetectable concentration within 7 to 14 days, depending on the dose and duration of treatment. Itraconazole mean total plasma clearance following intravenous administration is 278 mL/min. Itraconazole clearance decreases at higher doses due to saturable hepatic metabolism.[6]

Itraconazole is extensively metabolized in the liver, with CYP3A4 being the major enzyme that is involved in its metabolism. Itraconazole is also a potent inhibitor of CYP3A4; interactions with other drugs metabolized by the same enzyme have been observed. On the basis of the data reported, the following recommendations can be made with regard to clinical practice concerning therapy with itraconazole and concomitant medications.[9]

Monitoring of drug levels and/or effects, including side effects is recommended in patients treated with oral anticoagulants, antihuman-immunodeficiency-virus protease inhibitors, such as indinavir, certain antineoplastic agents such as busulphan, CYP3A4 metabolized calcium channel blockers such as dihydropiridines, certain immunosuppressive agents such as cyclosporin and tacrolimus, and digoxin, buspirone, alprazolam, intravenous midazolam and methylprednisolone. If necessary, doses of this medication should be reduced.[9]

Terfenadine, astemizole, cisapride, oral midazolam, triazolam, quinidine and CYP3A4 metabolized HMG-CoA (3-hydroxy-3-methyl-glutaryl-coenzyme A) reductase inhibitors such as simvastatin and lovastatin should not be used during treatment with itraconazole.[9]

Potent inhibitors of CYP3A4, such as clarithromycin and ritonavir can increase the bioavailability to itraconazole. Enzyme inducers such as phenytoin, phenobarbital, carbamazepine, rifabutin and rifampicin can decrease the exposure to itraconazole to such an extent that the efficacy of itraconazole may be largely reduced. Therefore, the combination of itraconazole with potent enzyme inducers should be avoided.[9]

Absorption of itraconazole from itraconazole capsules is impaired when the gastric acidity is reduced. Acid neutralizing medicines should be administered at least 2 hours after intake of itraconazole capsules. In patients with achlorhydria such as certain AIDS (acquired immunodeficiency syndrome) patients and patients on acid secretion suppressors it is advisable to administer itraconazole capsules with a cola beverage.

Pharmacokinetic studies have demonstrated the high tissue affinity of itraconazole as well as the persistence of itraconazole in the stratum corneum up to 4 weeks after discontinuation of therapy. These properties have provided a basis for further refinement of treatment with itraconazole capsules and have led to the development of short course regimens of treatment.[9]

2.3. Benefit/Risk Assessment

The international birth date for itraconazole[i] is 31 August 1987, based on first approval in Mexico. To date, itraconazole is registered in 111 countries worldwide. In India, itraconazole 100 mg capsules was approved on 20 July 1993 for the treatment of adult patients (age >18 years) in the following conditions:

- Gynecological indications: vulvovaginal candidosis
- Dermatological/mucosal/ophthalmological indications: dermatomycosis; pityriasis versicolor; oral candidosis; fungal keratitis
- Onychomycosis (caused by dermatophytes and/or yeasts)
- Systemic mycoses: systemic aspergillosis and candidosis; cryptococcosis (including cryptococcal meningitis); histoplasmosis; blastomycosis, porotrichosis; paracoccidiodomycosis; chromomycosis; and penicilliosis[9,21]

The safety of itraconazole capsules was evaluated in 8,499 subjects who participated in 107 open-label and double-blind clinical trials. The overall safety profile of itraconazole was similar for patients regardless of indication.[9,21]

For the indication related to this application, a total of 839 patients with *T. cruris or T. corporis* were randomized to 3 double-blind comparatives and 1 open trial. Out of 828 patients who were included in the intent-to-treat analyses, 463 patients were treated with 200 mg itraconazole for 1 week, 120 with 100 mg itraconazole for 2 weeks, while 78 patients received 250 mg terbinafine for 1 week and 167 received this dose for 2 weeks. As observed in these clinical trials, a treatment of 1 week with 200 mg itraconazole daily was effective in the treatment of *T. cruris or T. corporis*, with endpoint mycological cure ranging between 60% and 90%, and clinical response rates between 73% and 100%. Of the 11 excluded patients, four had been randomized to itraconazole 200 mg daily for 1 week. The overall incidence of adverse events reported during the trials was lowest (10%) with itraconazole 200 mg once daily as compared to the other treatment groups including placebo. One patient in the 200 mg itraconazole group had serious adverse event, but the same was attributed to concomitant medication and the patient recovered later. Discontinuation for adverse events occurred in 5 patients (1%) in the 200 mg itraconazole for 1-week group.[19]

Further, in these clinical trials, a total of 670 patients were included in the analysis of laboratory samples, comprising 68 patients on itraconazole 200 mg for 1 week, 272 patients on itraconazole 400 mg for 1 week, 51 and 68 on itraconazole 100 mg for 2 weeks and 4 weeks respectively, 50 patients on placebo and 161 patients on terbinafine. Blood samples were drawn at entry and after 1 week of treatment. Paired laboratory data (baseline and treatment) were available for 643 patients. Overall, there were no consistent changes pointing towards any specific toxicity associated to the dosage schedules under investigation (1-week 200 mg itraconazole): observed

[i] "Itraconazole" in the Benefit/Risk Assessment section refers to sponsor's itraconazole brand, SPORANOX, as all data in this section pertains to SPORANOX only.

changes were small, did not follow consistent patterns, and did not affect a specific parameter. The evaluation of the laboratory parameters did not indicate any specific toxicity of itraconazole.[19]

In conclusion, this evaluation indicates that itraconazole at daily dosages of 200 mg for 1 week, is safe and well tolerated for the treatment of *T. cruris or T. corporis*.[19]

A cumulative analysis of all spontaneously reported adverse drug experiences and individually documented side effects described in the scientific literature covering the period between 01 April 2012 to 31 March 2017 is available. Based on the 560,632,133 capsules (generic and non-generic) distributed in this period, the estimated exposure to itraconazole capsules are 23,760,124 treatment courses. During the same reporting period, 56 cases reporting events related to cardiac failure, 29 cases reporting events related to QT prolongation/ventricular arrhythmias, 76 cases reporting events related to other cardiac arrhythmias, 6 cases reporting events related to hepatic failure, 1 case of transitory deafness, 23 cases of peripheral neuropathy, 233 cases reporting events related to drug interaction were identified.[9]

In this real-world study, potential risks of treatment with itraconazole (eg, cardiac failure, hepatic failure, and hypersensitivity reactions) are being addressed in multiple ways:

- Exclusion criteria (Section 5.2) prevents the inclusion of participants with history of ventricular dysfunction such as congestive heart failure (CHF) or taking treatment for CHF, history of liver and/or renal impairment disease, history of any other serious co-morbid conditions, history of reactions to itraconazole or to any of the excipients, and baseline laboratory abnormalities.

- Exclusion of participants on certain concomitant medications (Section 5.2) that are CYP3A4 substrates as increased plasma concentrations of these drugs, caused by co-administration with itraconazole, may increase or prolong both therapeutic and adverse effects to such an extent that a potentially serious situation may occur.

- Lastly, exclusion of women of childbearing age not on any effective contraceptive precautions, pregnant and lactating women.

The likelihood of having a serious AE in the study is very small. Further, the study participants are patients who are receiving itraconazole as part of their clinical care. The only additional risks associated with participation in the study are:

a. Loss of time due to an additional visit to the clinic at the end of treatment.
b. The small risk of having a venous blood sample taken to measure plasma concentration of itraconazole.
c. The small risk of having skin scraping sample taken for potassium hydroxide (KOH) mount and culture and sensitivity test.

The benefit for the participants associated with this study can be summarized as below:

a. Information about the causative organism and plasma drug concentrations will likely improve their future care in case of treatment failure or reinfection.

Based on the available safety data in *T. cruris or T. corporis* and other disease indications, efficacy data in the earlier study, and proposed safety measures, the overall risk/benefit assessment of itraconazole in this protocol is acceptable.

More detailed information about the known and expected benefits and risks of itraconazole may be found in the SmPC.[20]

3. OBJECTIVES AND ENDPOINTS

Objectives	Endpoints
**Primary**	
• Estimate the proportion of participants prescribed itraconazole for *T. cruris* or *T. corporis* who have clinical response after 7 days of treatment	• Proportion of participants with clinical response (defined as "healed" or "markedly improved" based on the Investigator Global Evaluation tool of clinical improvement [see Appendix 10.2]) at the end of 7 days of treatment
**Secondary**	
• Estimate the proportion of participants prescribed itraconazole for *T. cruris* or *T. corporis* who have mycological response after 14 days of follow-up	• Mycological cure defined as both culture and microscopy negative
• Association of the clinical response at Day 7 with plasma drug concentrations of itraconazole and hydroxy-itraconazole	• Plasma concentrations of itraconazole and hydroxy-itraconazole during therapy
• Association of the clinical response at Day 7 with the baseline sensitivity pattern of causative fungi	• Baseline minimum inhibitory concentration (MIC)
• Estimate proportion of participants with clinical response after 14 days of follow-up. Estimate the extent to which clinically improved at Day 7 predicts clinically improved at Day 14	• Proportion of participants with clinical response (defined as “healed” or “markedly improved” based on the Investigator Global Evaluation tool of clinical improvement [see Appendix 10.2]) at the end of 14 days of follow-up
**Tertiary/Exploratory**	
• Evaluate the proportion of participants that come for follow-up after 7 days of treatment	• Proportion of participants coming for evaluation after 7 days of treatment

4. STUDY DESIGN

4.1. Overall Design

This is a real-world, prospective, non-randomized, open-label, multicenter, interventional, longitudinal pilot study to evaluate clinical outcomes and risk factors associated with clinical response in adult participants from India with *T. cruris* or *T. corporis* treated with itraconazole, including reference itraconazole, as part their clinical care. The interventions in this study will be

the systematic evaluation of clinical response, cultures of causative organism from the lesions and determination of plasma concentrations of itraconazole and hydroxy-itraconazole.

A target of 50 participants consisting of men and women between 18 to 60 years of age with *T. cruris* or *T. corporis* infection that have been prescribed itraconazole 200 mg daily will be enrolled in this study. Participants will be recruited from clinical practices of dermatologists in India. The study will be conducted at 4 to 5 large clinics of dermatologists across different regions of India and will include sites that have their own pharmacy that dispense generic itraconazole and reference itraconazole. A minimum of 6 participants treated with reference itraconazole will be included. After the inclusion of 12 participants in the study, the number of participants with reference itraconazole will be evaluated. If there are less than 3 participants receiving reference itraconazole a mitigation plan will be implemented with the inclusion of one more site with a pharmacy to increase the number of participants treated with reference itraconazole. Recruitment time for this pilot study is planned for 4 months. If after 4 months less than 50 participants in total have been recruited or less than 6 participants with reference itraconazole, the recruitment period will be extended up to a total of 6 months. The data available after a maximum of 6 months of recruitment will be used for analysis.

The study will consist of 3 phases: a screening phase; a 7-day open-label treatment phase; a 7-day observation phase; and end of study (EOS) visit at Day 14. The duration of individual participation will be approximately 15 days.

At baseline, all participants with *T. cruris* or *T. corporis* that have been prescribed itraconazole 200 mg daily by their treating physician as part of their clinical care and meet eligibility criteria will undergo a clinical evaluation of signs and symptoms using an Investigator Global Evaluation tool and skin scraping collection for KOH mount and culture and drug sensitivity. The brand and strength of (100 mg or 200 mg) itraconazole will be determined by the treating physician and local pharmacies where the study drug is dispensed. Participants will receive their regular clinical care at the discretion of their treating physician and will enter the open-label, treatment phase and take 200 mg of itraconazole daily with a full meal on Day 1 through Day 7. Clinical response using the Investigator Global Evaluation tool (see Appendix 10.2) will be done at Day 7 (first assessment visit) and on Day 14 (second assessment visit). At first assessment visit on Day 7, participants will undergo collection of 3 blood samples to determine plasma concentration of itraconazole and its main metabolite, hydroxy-itraconazole. At the EOS visit on Day 14, skin scraping will be collected for KOH mount and culture. Treatment duration will be at the discretion of the physician. Only those participants that continue receiving itraconazole at the second assessment visit on Day 14 will undergo collection of 3 blood samples to determine plasma concentration of itraconazole and hydroxy-itraconazole.

For further details on study procedures, see Schedule of Activities (SoA) in Section 1.3.

A diagram (Figure 1) of the study design is provided in Section 1.2, Schema.

4.2. Scientific Rationale for Study Design

Participant Population

To ensure the inclusion of participants who receive the standard of care in India, the study population comprises of adults with *T. cruris* or *T. corporis* who have been prescribed itraconazole by their treating physician as part of their clinical care. The participants will be recruited from 4 to 5 large clinics of dermatologists across different regions of India, and will include sites that have their own pharmacies that dispense itraconazole and reference itraconazole. A minimum of 6 participants treated with reference itraconazole will be included or the mitigation plan of study extension will be implemented (see Section 4.1).

Dosage and Administration

Since participants that receive standard of care will be included, the dose and administration will be selected at the discretion of the treating physician. The recommended oral itraconazole dosage in India[21] for dermatomycosis infections is 200 mg once daily for 7 days or 100 mg once daily for 15 days. Only participants that are prescribed 200 mg daily will be included and will be followed for up to 14 days.

Pharmacokinetic Assessments

Blood samples are to be obtained at specified times after study drug dosing for measurement of plasma concentrations of itraconazole and its main metabolite, hydroxy-itraconazole. These concentrations will be used to explore the relationship between plasma concentrations of itraconazole and/or hydroxy-itraconazole and clinical outcomes after oral administration of multiple doses of various itraconazole-containing products in participants. These data will also be used to evaluate the overall distribution of plasma concentrations of itraconazole and hydroxy-itraconazole in participants who receive itraconazole as different products.

Clinical Efficacy Assessments

Clinical efficacy will be evaluated with the Clinical Assessment Tools. The investigator will use the Clinical Evaluation Tool to assess the severity of signs and symptoms with a total score from 0 to 18 at each visit (baseline, at Day 7, and at Day 14). The total scores at Day 7 and at Day 14 compared with baseline scores will be used to define the percentage of clinical improvement. The percentage of clinical improvement will be used to classify the clinical efficacy using the Investigator Global Evaluation Tool (Table 2 of Appendix 10.2). A score from 1 to 5 will be assigned at Day 7 and at Day 14 based on the percentage of clinical improvement (Table 2). Clinical Response will be defined as having scores 1 or 2 (“healed” or “markedly improved”).

Mycological evaluation will be done at baseline and at the end of 14 days follow-up. A participant will be defined as mycologically cured upon negative culture and microscopy result at the end of follow-up.

Microbiological Assessments

Skin scraping for KOH, culture and sensitivity analysis will be performed at baseline and for KOH and culture at Day 14 (EOS visit). Potassium hydroxide will confirm the diagnosis of *Tinea* at

baseline. Culture and sensitivity results will be used to evaluate the association of baseline resistance of itraconazole and clinical outcomes.

4.2.1. Study-Specific Ethical Design Considerations

Potential participants will be fully informed of the risks and requirements of the study and, during the study, participants will be given any new information that may affect their decision to continue participation. They will be told that their consent to participate in the study is voluntary and may be withdrawn at any time with no reason given and without penalty or loss of benefits to which they would otherwise be entitled. Only participants who are fully able to understand the risks, benefits, and potential adverse events of the study, and provide their consent voluntarily will be enrolled.

Only participants that have been prescribed itraconazole 200 mg daily for the treatment of *T. cruris* or *T. corporis* by their treating physician will be included. There are no additional risks associated with the study drug as they are risks associated with participants' routine clinical care.

The primary ethical concern is the risk associated with venipuncture and multiple blood sample collection. A small number of blood samples will be taken from each participant to minimize discomfort to the participants while obtaining sufficient drug exposure data for correlation with clinical outcomes.

The total blood volume to be collected from each participant during the study is less than a standard 500 mL blood donation according by the American Red Cross.[1]

4.3. Justification for Dose

The approved dosage of itraconazole for *T. cruris or T. corporis* is 100 mg daily for 15 days or 200 mg daily for 7 days. The dose will be selected at the discretion of the treating physician. Only participants that receive itraconazole 200 mg daily will be included to facilitate interpretation of the plasma concentration of itraconazole and its main metabolite, hydroxy-itraconazole.

4.4. End of Study Definition

A participant can be considered to have completed the study if they have completed at least 7-day treatment and clinical evaluations at baseline, at Day 7, and at Day 14.

The EOS is considered as the last visit for the last participant in the study. The final data from the study site will be sent to the sponsor (or designee) after completion of the final participant visit at that study site, in the time frame specified in the Clinical Trial Agreement.

5. STUDY POPULATION

Screening for eligible participants will be performed within 3 days before administration of the itraconazole. Refer to Section 5.4, Screen Failures for conditions under which the repeat of any screening procedures are allowed.

The inclusion and exclusion criteria for enrolling participants in this study are described below. If there is a question about these criteria, the investigator must consult with the appropriate sponsor

representative and resolve any issues before enrolling a participant in the study. Waivers are not allowed.

For a discussion of the statistical considerations of participant selection, refer to Section 9.2, Sample Size Determination.

5.1. Inclusion Criteria

Each potential participant must satisfy all of the following criteria to be enrolled in the study:

1. Clinically diagnosed with *T. cruris* or *T. corporis* with or without history of treatment

2. Are prescribed itraconazole at 200 mg/day orally for the treatment of *T. cruris* or *T. corporis* and taken once a day

3. 18 to 60 years of age (both inclusive)

4. Must sign an informed consent form (ICF) indicating that he or she understands the purpose of, and procedures required for, the study and is willing to participate in the study

5. A woman of childbearing potential must have a negative highly sensitive serum (β-human chorionic gonadotropin [β-hCG]) at screening on Day -3 to 0 before start of study drug.

6. A woman must be (as defined in Section 10, Appendix 10.3, Contraceptive Guidance and Collection of Pregnancy Information)

    a. Not of childbearing potential

    b. Of childbearing potential and:

    Practicing a highly effective method of contraception (failure rate of <1% per year when used consistently and correctly) and agrees to remain on a highly effective method while receiving study drug and until 30 days after last dose (ie, the end of relevant systemic exposure). Examples of highly effective methods of contraception are located in Appendix 10.3, Contraceptive and Barrier Guidance and Collection of Pregnancy Information.

    Typical use failure rates may differ from those when used consistently and correctly

7. A male participant must wear a condom when engaging in any activity that allows for passage of ejaculate to another person. A male participant must agree not to donate sperm for the purpose of reproduction during the study and for a minimum 90 days after receiving the last dose of study drug.

5.2. Exclusion Criteria

Any potential participant who meets any of the following criteria will be excluded from participating in the study:

1. History of ventricular dysfunction such as CHF or receiving treatment for CHF, liver or renal insufficiency; significant cardiac, vascular, pulmonary, gastrointestinal, endocrine, neurologic, hematologic, rheumatologic, psychiatric, or metabolic disturbances at any time prior to the start of study

2. Known achlorhydria or on treatment of gastric acidity

3. Presence of other dermatoses, e.g. psoriasis, seborrhoeic or atopic dermatitis

4. Infected with organism with known or established resistance to itraconazole

5. Co-existing fungal infection of other body area

6. Known allergies, hypersensitivity, or intolerance to itraconazole or its excipients or to any other azole[21]

7. Pregnant, or breastfeeding women

8. Women of childbearing potential not on any effective contraceptive precautions or planning to become pregnant while enrolled in this study or within 2 weeks after the last dose of study drug

9. Taking drugs that are CYP3A4 substrates (see Section 6.5, Concomitant Therapy before the planned first dose of study drug for disallowed therapies)

10. Received oral itraconazole within 14 days before screening

11. Use of other systemic antifungal or corticosteroid therapy unless such therapy has been discontinued for at least 30 days prior to the start of therapy

12. Use of topical antifungal or corticosteroid therapy within 14 days before screening

13. If at criteria of the treating physician there is a proven or suspected infection with *Fusarium* species, mucorales or other agents considered not susceptible to itraconazole

14. Received an investigational drug (including investigational vaccines) or used an invasive investigational medical device within 3 months before the planned first dose of study drug or is currently enrolled in an investigational study

15. Plans to father a child while enrolled in this study or within 2 weeks after the last dose of study drug

16. Any condition for which, in the opinion of the investigator, participation would not be in the best interest of the participant (eg, compromise the well-being) or that could prevent, limit, or confound the protocol-specified assessments

NOTE: Investigators should ensure that all study enrollment criteria have been met at screening. If a participant's clinical status changes (including any available laboratory results or receipt of additional medical records performed at the discretion of the treating physician based on standard of care) after screening but before the first dose of study drug is given such that if he or she no longer meets all eligibility criteria, then the participant should be excluded from participation in the study. Section 5.4, Screen Failures, describes options for retesting. The required source documentation to support meeting the enrollment criteria are noted in Appendix 10.4, Regulatory, Ethical, and Study Oversight Considerations.

5.3. Lifestyle Considerations

Potential participants must be willing and able to adhere to the following lifestyle restrictions during the course of the study to be eligible for participation:

1. Refer to Section 6.5, Concomitant Therapy for details regarding prohibited and restricted therapy during the study

2. Agree to follow all requirements that must be met during the study as noted in the Inclusion and Exclusion Criteria (eg, contraceptive requirements)

5.4. Screen Failures

Participant Identification, Enrollment, and Screening Logs

The investigator agrees to complete a participant identification and enrollment log to permit easy identification of each participant during and after the study. This document will be reviewed by the sponsor study-site contact for completeness.

The participant identification and enrollment log will be treated as confidential and will be filed by the investigator in the study file. To ensure participant confidentiality, no copy will be made. All reports and communications relating to the study will identify participants by participant identification and date of birth (as allowed by local regulations). In cases where the participant is not enrolled into the study, the date seen and date of birth (as allowed by local regulations) will be used.

Individuals who do not meet the criteria for participation in this study (screen failure) may be rescreened. Individuals who previously failed screening as a result of positive pregnancy test may be retested. Rescreened participants should be assigned the same participant number as for the initial screening. A rescreened participant can be enrolled only after approval from Medical Monitor of this study.

6. STUDY INTERVENTION

6.1. Study Interventions Administered

In clinical trials, the study drug is usually the intervention. In this study, the study drug is not part of the intervention.

Itraconazole capsules prescribed by treating physicians will be dispensed by the pharmacies of their choice. Participants will be instructed to take their prescribed dose of itraconazole orally each day after a full meal. The dose will be 200 mg capsules or 2, 100 mg capsules once daily taken on Day 1 after screening through Day 7. Total duration of treatment will be decided at the discretion of the physician as part of the subject's clinical care and standard of practice.

Participants will be asked to maintain a dose-time diary in which they will record the time and date of the dose taken each day. All study drug must be stored as described on the drug package and kept out of reach of children.

Refer to the IB/Prescribing Information for a list of excipients.

6.2. Preparation/Handling/Storage/Accountability

Not applicable as the sponsor will not be providing study drug.

6.3. Measures to Minimize Bias: Randomization and Blinding

As this is an open-label study, blinding procedures are not applicable.

6.4. Study Intervention Compliance

Study drug will be self-administered by participants starting after screening from Day 1 through Day 6. On Day 7 participants will be administered the dose at the clinic. If at the discretion of the treating physician participants are still receiving itraconazole at visit on Day 14, the Day 14 dose will also be administrated at the clinic. Prescribed itraconazole will be dispensed to participants after screening either at pharmacies within the dermatologists' clinic or from outside pharmacies.

Participants will be instructed to bring in their study drug in their original containers/package as dispensed to them to assess compliance and to record the brand of itraconazole that they received. The number of dispensed capsules will be recorded and compared with the number of capsules returned at study visits on Day 7 and Day 14. At the screening visit, participants will receive instructions on compliance with study drug administration and dose-time diaries for recording time of when study drug was taken. During the course of the study, the investigator or designated study-site personnel will be responsible for providing additional instruction to reeducate any participant who is not compliant with taking the study drug.

6.5. Concomitant Therapy

Prestudy therapies administered up to 14 days before first dose of study drug must be recorded at screening.

Concomitant therapies must be recorded throughout the study beginning with start of the first dose of study drug to the last dose of study drug. Concomitant therapies should also be recorded beyond 14 days only in conjunction with serious adverse events that meet the criteria outlined in Serious Adverse Events in Section 8.3.1, Time Period and Frequency for Collecting Adverse Event and Serious Adverse Event Information.

All therapies (prescription or over-the-counter medications, including vaccines, vitamins, herbal supplements; non-pharmacologic therapies such as electrical stimulation, acupuncture, special diets, exercise regimens) different from the study drug must be recorded in the Case Report Forms (CRFs). Recorded information will include a description of the type of therapy, duration of use, dosing regimen, route of administration, and indication. Modification of an effective preexisting therapy should not be made for the explicit purpose of entering a participant into the study.

6.5.1. Prohibited Medication

The use of the following drugs is not permitted concomitantly with itraconazole administration through Day 14:

- All other antifungal agents (including topical antifungal agents applied to skin or vagina or systemic medications)
- Topical or systemic steroids
- CYP3A4-metabolized drugs/P-glycoprotein-transported drugs (including but not limited to cisapride, pimozide, quinidine, dofetilide, levacetylmethadol, terfenadine, astemizole, mizolastine, bepridil, sertindole, HMG-CoA reductase inhibitor [eg, simvastatin, lovastatin], oral midazolam, triazolam, ergot alkaloids [eg, dihydroergotamine, ergonovine, ergotamine, methylergonovine] and nisoldipine)
- Any investigational drugs
- Any antipruritic medications interacting with itraconazole including but not limited to astemizole, bilastine, ebastine, rupatadine, mizolastine, and terfenadine
- Drugs that reduce gastric acidity

6.6. Dose Modification

Any dose/dosage adjustment of itraconazole should be overseen by medically-qualified study-site personnel (principal or subinvestigator unless an immediate safety risk appears to be present).

6.7. Intervention After the End of the Study

There will be no study drug following the end of the study.

7. DISCONTINUATION OF STUDY INTERVENTION AND PARTICIPANT DISCONTINUATION/WITHDRAWAL

7.1. Discontinuation of Study Intervention

A participant's study drug must be discontinued if:

- The investigator believes that for safety reasons or tolerability reasons (eg, adverse event) it is in the best interest of the participant to discontinue study drug
- The participant becomes pregnant. Refer to Appendix 10.3, Contraceptive Guidance and Collection of Pregnancy Information
- The participant has an anaphylactic reaction during or following administration of study agent
- The participant withdraws consent for administration of itraconazole

If a participant discontinues study drug for any reason before the end of the treatment period, assessments should be obtained as specified in SoA (Section 1.3). If the reason for discontinuation of itraconazole is withdrawal of consent then no additional assessments are allowed.

7.2. Participant Discontinuation/Withdrawal From the Study

A participant will not be automatically withdrawn from the study if they have to discontinue study drug before the end of the drug regimen.

A participant will be withdrawn from the study for any of the following reasons:

- Lost to follow-up
- Withdrawal of consent
- Death
- Noncompliance defined as participant failed to take 80% of study drug
- The study investigator or the sponsor, for any reasons, stops the study or stops the participant's participation in the study

When a participant withdraws before completing the study, the reason for withdrawal is to be documented in the CRF and in the source document. Study drug prescribed to the withdrawn participant may not be assigned to another participant. Additional participants will be entered to ensure the protocol-specified number of participants complete the study. If a participant discontinues study drug and withdraws from the study before the end of the end-of-intervention assessments should be obtained. If the reason for withdrawal from the study is withdrawal of consent, then no additional assessments are allowed.

7.3. Lost to Follow-up

If a participant is lost to follow-up, every reasonable effort must be made by the study-site personnel to contact the participant and determine the reason for discontinuation/withdrawal. The measures taken to follow-up must be documented. Refer to Section 7.2, Participant Discontinuation/Withdrawal From the Study.

8. STUDY ASSESSMENTS AND PROCEDURES

Overview

All participants will receive clinical care at the discretion of their treating physicians as per the standard of practice.

The SoA (Section 1.3) summarizes the frequency and timing of efficacy and safety measurements applicable to this study.

Assessment of clinical response to treatment will be done using an Investigator Global Evaluation tool based on signs and symptoms and clinical improvement scores (See Section 8.1, Efficacy Assessments and Appendix 10.2) at follow-up visits.

Skin scraping will be collected at baseline for KOH mount, culture and drug sensitivity and on Day 14 for KOH mount and culture (see Table 4 of Appendix 10.6).

During screening, each participant will be provided with a dose-time diary for recording time of when study drug was taken. Study-site personnel will train the participants on how to use the diary, including instructions to capture the data according to the study design and not to wait until the study-site visit to record information.

A serum pregnancy test will be performed at screening. Blood samples to determine plasma concentration of itraconazole and its main metabolite, hydroxy-itraconazole will also be collected on Day 7 and Day 14 (see Table 3 and Table 4 of Appendix 10.6).

The total blood volume for the study is approximately 42 mL (36 mL for plasma drug concentration and 6 mL for pregnancy testing [women only]).

For each participant, the maximum amount of blood drawn from each participant in this study will not exceed 500 mL.

Repeat or unscheduled samples may be taken for safety reasons or for technical issues with the samples.

Study-Specific Materials

The investigator will be provided with the following supplies:

- SmPC/India Prescribing Insert
- Pharmacy manual/study-site investigational product and procedures manual
- Laboratory manual
- Sample ICF
- Participant dose-time diaries
- CRFs

8.1. Efficacy Assessments

Clinical efficacy will be evaluated with the Clinical Assessment Tools. The investigator will use the Clinical Evaluation Tool to assess the severity of signs and symptoms with a total score from 0 to 18 at each visit (baseline, at Day 7, and at Day 14). The total scores at Day 7 and at Day 14 compared with baseline scores will be used to define the percentage of clinical improvement. The percentage of clinical improvement will be used to classify the clinical efficacy using the Investigator Global Evaluation Tool (Table 2 of Appendix 10.2). A score from 1 to 5 will be assigned at Day 7 and at Day 14 based on the percentage of clinical improvement (Table 2).

Clinical Response will be defined as having scores 1 or 2 (“healed” or “markedly improved”).

Plasma concentrations of itraconazole and hydroxy-itraconazole collected from venous blood samples will be assessed against clinical outcomes.

Skin scrapings collection for culture will be conducted at baseline and at Day 14 to assess mycological cure.

Skin scraping for culture and sensitivity analysis will be collected to evaluate baseline resistance to itraconazole and its association with clinical outcomes. Antifungal sensitivity testing will be carried out with a micro dilution method according to the Clinical and Laboratory Standards Institute (CLSI) M27 guidelines. The minimum inhibitory concentration (MIC) of itraconazole toward fungal pathogens from each individual participant before study drug will be determined.

8.2. Safety Assessments

Clinical evaluation of adverse events will be performed at discretion of the treating physician, based on the standard of care. Adverse events will be reported and followed by the investigator as specified in Section 8.3, Adverse Events and Serious Adverse Events and Appendix 10.5, Adverse Events: Definitions and Procedures for Recording, Evaluating, Follow-Up, and Reporting.

Any clinically relevant changes occurring during the study must be recorded on the Adverse Event section of the CRF.

Any clinically significant abnormalities persisting at the end of the study/early withdrawal will be followed by the investigator until resolution or until a clinically stable condition is reached.

A pregnancy test will be performed at screening.

Anticipated events will not be recorded and reported as there are no anticipated events associated with *T. cruris* or *T. corporis*.

8.2.1. Clinical Safety Laboratory Assessments

Clinical safety laboratory assessments will be done at the discretion of the treating physician as part of the standard practice, except for a protocol-required serum pregnancy test at screening (see Table 3 of Appendix 10.6). The investigator must review the laboratory results, document this

review, and record any clinically relevant changes occurring during the study in the adverse event section of the CRF. The laboratory reports must be filed with the source documents.

8.3. Adverse Events and Serious Adverse Events

Timely, accurate, and complete reporting and analysis of safety information from clinical studies are crucial for the protection of participants, investigators, and the sponsor, and are mandated by regulatory agencies worldwide. The sponsor has established Standard Operating Procedures in conformity with regulatory requirements worldwide to ensure appropriate reporting of safety information; all clinical studies conducted by the sponsor or its affiliates will be conducted in accordance with those procedures.

Adverse events will be reported by the participant (or, when appropriate, by a caregiver, surrogate, or the participant's legally acceptable representative) for the duration of the study.

Anticipated events will not be recorded and reported as there are no anticipated events associated with *T. cruris* or *T. corporis*.

For further details on adverse events and serious adverse events (Definitions and Classifications; Attribution Definitions; Severity Criteria; Special Reporting Situations; Procedures) as well as product quality complaints, refer to Appendix 10.5, Adverse Events: Definitions and Procedures for Recording, Evaluating, Follow-Up, and Reporting.

8.3.1. Time Period and Frequency for Collecting Adverse Event and Serious Adverse Event Information

All Adverse Events

All adverse events and special reporting situations, whether serious or non-serious, will be reported from the time a signed and dated ICF is obtained until completion of the participant's last study-related procedure, which may include contact for follow-up of safety. Serious adverse events, including those spontaneously reported to the investigator within 30 days after the last dose of study drug, must be reported using the Serious Adverse Event Form. The sponsor will evaluate any safety information that is spontaneously reported by an investigator beyond the time frame specified in the protocol.

Serious Adverse Events

All serious adverse events occurring during the study must be reported to the appropriate sponsor contact person by study-site personnel within 24 hours of their knowledge of the event.

Information regarding serious adverse events will be transmitted to the sponsor using the Serious Adverse Event Form and Safety Report Form of the CRF, which must be completed and reviewed by a physician from the study site, and transmitted to the sponsor within 24 hours. The initial and follow-up reports of a serious adverse event should be transmitted electronically or by facsimile (fax).

8.3.2. Method of Detecting Adverse Events and Serious Adverse Events

Care will be taken not to introduce bias when detecting adverse events or serious adverse events. Open-ended and nonleading verbal questioning of the participant is the preferred method to inquire about adverse event occurrence.

Solicited Adverse Events

Solicited adverse events are predefined local and systemic events for which the participant is specifically questioned.

Unsolicited Adverse Events

Unsolicited adverse events are all adverse events for which the participant is not specifically questioned.

8.3.3. Follow-up of Adverse Events and Serious Adverse Events

Adverse events, including pregnancy, will be followed by the investigator as specified in Appendix 10.5, Adverse Events: Definitions and Procedures for Recording, Evaluating, Follow-up, and Reporting.

8.3.4. Regulatory Reporting Requirements for Serious Adverse Events

The sponsor assumes responsibility for appropriate reporting of adverse events to the regulatory authorities. The sponsor will also report to the investigator (and the head of the investigational institute where required) all suspected unexpected serious adverse reactions (SUSARs).

8.3.5. Pregnancy

All initial reports of pregnancy in female participants or partners of male participants must be reported to the sponsor by the study-site personnel within 24 hours of their knowledge of the event using the appropriate pregnancy notification form. Any participant who becomes pregnant during the study must be promptly withdrawn from the study.

8.4. Treatment of Overdose

For this study, any dose of itraconazole greater than 400 mg within a 24-hour time period will be considered an overdose.

In the event of an overdose, the investigator or treating physician should:

- Closely monitor the participant for AE/SAE and laboratory abnormalities until itraconazole can no longer be detected systemically (at least 14 days)
- Document the quantity of the excess dose as well as the duration of the overdosing in the CRF

Decisions regarding dose interruptions or modifications will be made by the investigator in consultation with the Medical Monitor based on the clinical evaluation of the participant.

8.5. Pharmacokinetics

Blood samples will be used to evaluate plasma drug concentrations of itraconazole and its main metabolite, hydroxy-itraconazole. Blood collected may additionally be used to evaluate safety or efficacy aspects that address concerns arising during or after the study period. Genetic analyses will not be performed on these blood samples. Participant confidentiality will be maintained.

8.5.1. Sample Collection and Handling

The actual dates and times of sample collection must be recorded in the CRF or laboratory requisition form. If blood samples are collected via an indwelling cannula, an appropriate amount (1 mL) of serosanguineous fluid slightly greater than the dead space volume of the lock will be removed from the cannula and discarded before each blood sample is taken. After blood sample collection, the cannula will be flushed with 0.9% sodium chloride, United States Pharmacopeia (USP) (or equivalent) and charged with a volume equal to the dead space volume of the lock. If a mandarin (obturator) is used, blood loss due to discard is not expected.

Blood sample collection will occur 24 hours (±2 hours) after previous dose (Day 6, Day 13) and 2 and 4.5 hours ±10 minutes after the last dose (Day 7, Day 14). Refer to the SoA (Sections 1.3) for the timing and frequency of all sample collections.

Instructions for the collection, handling, storage, and shipment of samples are found in the laboratory manual that will be provided. Collection, handling, storage, and shipment of samples must be under the specified conditions, and where applicable, controlled temperature conditions as indicated in the laboratory manual.

8.5.2. Analytical Procedures

Pharmacokinetics

Plasma samples will be analyzed to determine concentrations of itraconazole and hydroxy-itraconazole using a validated, specific, and sensitive high-performance liquid chromatography (HPLC) method with tandem mass spectrometric detection (LC MS/MS) by or under the supervision of the sponsor.

8.6. Pharmacodynamics

Not applicable

8.7. Genetics

Not applicable

8.8. Biomarkers

Not applicable

9. STATISTICAL CONSIDERATIONS

Statistical analysis will be done by the sponsor or under the authority of the sponsor. A general description of the statistical methods to be used to analyze the plasma concentrations of

itraconazole and its main metabolite, hydroxy-itraconazole and safety data is outlined below. Specific details will be provided in the Statistical Analyses, Section 9.4.

9.1. Statistical Hypotheses

All analyses will be descriptive. Although no hypothesis testing will be performed, the point estimate and the corresponding 95% Confidence Interval for the efficacy endpoints will be provided.

Data will be summarized using descriptive statistics. Continuous variables will be summarized using the number of observations, mean, standard deviation, median, and range as appropriate. Categorical variables will be summarized using the number of observations and the percentages.

9.2. Sample Size Determination

This study is a pilot study and the sample size was not calculated based on power, rather it was selected to ensure a minimum number of participants with reference itraconazole, (at least 6) out of 50 participants total.

After the inclusion of 12 participants in the study the number of participants with reference itraconazole will be evaluated. If there are less than 3 participants receiving SPORANOX a mitigation plan will be implemented with the inclusion of one more site with pharmacy to increase the number of participants treated with reference itraconazole.

9.3. Populations for Analyses

The analysis populations for this study are defined as the following:

All participants are defined as those participants who have enrolled in the study.

The pharmacokinetics (PK) population includes all participants that have at least one set of blood samples drawn at Day 7 to evaluate plasma drug concentrations of itraconazole and its main metabolite, hydroxy-itraconazole.

The safety population includes all participants that have received at least 1 administration of study drug.

A participant is considered to have completed the study if he/she have completed the 7-day treatment regime and had clinical evaluations at baseline, 7 days and at 14 days.

For purposes of analysis, the following populations are defined:

Population	Description
PK Population	Includes participants that have at least one set of blood samples drawn at Day 7
Efficacy population (intent-to-treat population [ITT])	Includes participants that completed the study if they have completed the 7-day treatment regime and had clinical evaluations at baseline and 7 days. Participants will be analyzed according to the study drug they are assigned.
Safety population	Includes participants that have received at least 1 administration of study drug. Participants will be analyzed according to the study drug they actually received.
Completer	Includes participants that completed the study if they have completed the 7-day treatment regime and had clinical evaluations at baseline, 7 days and at 14 days

9.4. Statistical Analyses

All analyses will be descriptive and no hypothesis testing will be performed.

9.4.1. Efficacy Analyses

Efficacy analyses will include primary endpoint (clinical response at Week 1) as well as all secondary endpoints. All efficacy analyses will be performed on the efficacy population (ITT).

Data will be summarized using descriptive statistics. Variables will be summarized using the number of observations, mean, standard deviation, median, and range as appropriate.

Primary Endpoint

The primary efficacy endpoint is defined as the estimated proportion of participants who have clinical response ("healed" or "markedly improved" of the Investigator Global Evaluation Tool of clinical improvement) after 7 days of treatment. The primary analysis will be performed on participants that have completed the 7-day treatment regime and had clinical evaluations at 7 days.

Major Secondary Endpoints

Major secondary endpoints include:

- Estimate the proportion of participants prescribed oral itraconazole who have mycological response after 14 days of follow-up
- Association of the clinical outcome with plasma concentrations of itraconazole and hydroxy-itraconazole. Plasma concentration data will be summarized by treatment (itraconazole or reference itraconazole) sampling time and analyte (see Section 9.4.3). The relationship between efficacy and plasma drug concentration will be explored graphically.
- Association of the clinical outcome with baseline MIC of causative fungi
- Estimation of the proportion of participants with clinical response after 14 days of follow-up. Estimate the extent to which clinically improved at Day 7 predicts clinically improved at Day 14

Tertiary/Exploratory Endpoints

Tertiary/exploratory endpoint will evaluate the proportion of patients that come for follow-up after 7 days of treatment.

9.4.2. Safety Analyses

Adverse Events

The verbatim terms used in the CRF by investigators to identify adverse events will be coded using the Medical Dictionary for Regulatory Activities (MedDRA). Treatment-emergent adverse events are adverse events with onset during the treatment phase or that are a consequence of a pre-existing condition that has worsened since baseline. All reported adverse events will be included in the analysis. For each adverse event, the percentage of participants who experience at least 1 occurrence of the given event will be summarized.

Summaries, listings, datasets, or participant narratives may be provided, as appropriate, for those participants who die, who discontinue drug due to an adverse event, or who experience a severe or a serious adverse event.

Physical Examination

Descriptive statistics of changes from baseline will be summarized at each scheduled time point.

Physical examination findings will be summarized at each scheduled time point. Descriptive statistics will be calculated at baseline and for observed values and changes from baseline at each scheduled time point. Frequency tabulations of the abnormalities will be made.

9.4.3. Pharmacokinetic Analyses

Data will be listed for all participants with available plasma concentrations for itraconazole and reference itraconazole. Participants will be excluded from the analysis if their data do not allow for accurate pharmacokinetic assessment (eg, incomplete administration of the study drug; missing information of dosing and sampling times; concentration data not sufficient for PK parameter calculation).

All concentrations below the lowest quantifiable concentration or missing data will be labeled as such in the concentration database. All participants and samples excluded from the analysis will be clearly documented in the study report.

Descriptive statistics, including arithmetic mean, standard deviation, coefficient of variation, median, minimum, and maximum will be determined for each sampling time and for any derived PK parameters of itraconazole and hydroxy-itraconazole.

9.5. Interim Analysis

There is no interim analysis planned for this study.

10. SUPPORTING DOCUMENTATION AND OPERATIONAL CONSIDERATIONS

10.1. Appendix 1: Abbreviations and Trademarks

CHF	congestive heart failure
CRFs	case report form(s) (paper or electronic as appropriate for this study)
EOS	end of study
eDC	electronic data capture
GCP	Good Clinical Practice
IB	Investigator's Brochure
ICF	informed consent form
ICH	International Conference on Harmonisation
IEC	Independent Ethics Committee
IRB	Institutional Review Board
KOH	potassium hydroxide
MIC	minimum inhibitory concentration
PK	pharmacokinetic(s)
PQC	Product Quality Complaint
SmPC	Summary of Product Characteristics
species	spp.
SoA	Schedule of Activities

10.2. Appendix 2: Clinical Assessment Tool

The Clinical Assessment Tool was used in the development program of itraconazole.[2,3,4,23] A total of 467 participants were included in 4 clinical trials that evaluated the use of itraconazole for the treatment of *T. cruris* or *T. corporis*. The clinical outcomes in these studies were evaluated with this methodology.

The Clinical Assessment includes 2 tools, the Clinical Evaluation tool (Table 1) to rate the severity of signs and symptoms at the established visits and 2. The Investigator Global Evaluation tool (Table 2) to classify the clinical response based on the percentage of clinical improvement from baseline.

Table 1: Clinical Evaluation Tool: Signs and Symptoms

Signs and Symptoms	Rating
Desquamation	Absent=0<br>Mild=1<br>Moderate=2<br>Severe=3
Erythema and Pruritus	
Exudation or incrustation	
Infiltration	
Maceration	
Vesiculation or Pustules	

Assessment of clinical response to treatment is based on the following rating scale:

Table 2: Investigator Global Evaluation Tool: Clinical Response

Global Evaluation	Score	Clinical Definition
Healed	1	Absence of signs and symptoms
Markedly Improved	2	≥50% clinical improvement
Considerable Residual Lesion	3	<50% clinical improvement
No Change	4	Same as baseline
Worse	5	Deterioration from baseline

Methodology of Clinical Assessment

The investigator will use the Clinical Evaluation Tool to score at baseline the following signs and symptoms as absent (=0), mild (=1), moderate (=2), or severe (=3): (Table 1): desquamation, erythema and pruritus, exudation or incrustation, infiltration, maceration, vesiculation or pustules. The total baseline clinical score will be the sum of all scores. Total scores of all signs and symptoms in each participant will range from 0 to 18. At the end of 7 days of treatment and 14 days follow-up, clinical signs and symptoms total scores will be recorded by the investigator. The total

scores at Day 7 and at Day 14 compared with baseline scores will be used to define the percentage of clinical improvement. The percentage of clinical improvement will be used to classify clinical efficacy using the Investigator Global Evaluation Tool (Table 2 of Appendix 10.2). A score from 1 to 5 will be assigned at Day 7 and at Day 14 based on the percentage of clinical improvement (Table 2).

Clinical Response will be defined as having scores 1 or 2 after 7 days of treatment ("healed" or "markedly improved") for the primary endpoint and after 14 days of treatment for the secondary endpoint.

10.3. Appendix 3: Contraceptive and Barrier Guidance and Collection of Pregnancy Information

Participants must follow contraceptive measures as outlined in Section 5.1, Inclusion Criteria. Pregnancy information will be collected and reported as noted in Section 8.3.5, Pregnancy and Appendix 10.5 Adverse Events: Definitions and Procedures for Recording, Evaluating, Follow-up, and Reporting.

Definitions

Woman of Childbearing Potential (WOCBP)

A woman is considered fertile following menarche and until becoming postmenopausal unless permanently sterile (see below).

Woman Not of Childbearing Potential

- **premenarchal**
  A premenarchal state is one in which menarche has not yet occurred.
- **postmenopausal**
  A postmenopausal state is defined as no menses for 12 months without an alternative medical cause. A high follicle stimulating hormone (FSH) level (>40 IU/L or mIU/mL) in the postmenopausal range may be used to confirm a postmenopausal state in women not using hormonal contraception or hormonal replacement therapy (HRT), however in the absence of 12 months of amenorrhea, a single FSH measurement is insufficient. If there is a question about menopausal status in women on HRT, the woman will be required to use one of the non-estrogen-containing hormonal highly effective contraceptive methods if she wishes to continue HRT during the study.
- **permanently sterile**
  Permanent sterilization methods include hysterectomy, bilateral salpingectomy, bilateral tubal occlusion/ligation procedures, and bilateral oophorectomy.

Note: If the childbearing potential changes after start of the study (eg, a premenarchal woman experiences menarche) or the risk of pregnancy changes (eg, a woman who is not heterosexually active becomes active), a woman must begin a highly effective method of contraception, as described throughout the inclusion criteria.

If reproductive status is questionable, additional evaluation should be considered.

As noted in Inclusion Criterion 5, study participants who are women of childbearing potential must be practicing a highly effective method of contraception and remain on a highly effective method while receiving study drug and until 30 days after last dose. Examples of highly effective methods of contraception are provided below; however, the method selected must meet local/regional regulations/guidelines for highly effective contraception.

Examples of Contraceptives

**EXAMPLES OF CONTRACEPTIVES[a] ALLOWED DURING THE STUDY INCLUDE:**
**USER INDEPENDENT**<br>**Highly Effective Methods That Are User Independent** *Failure rate of ≤1% per year when used consistently and correctly.*
• Implantable progestogen-only hormone contraception associated with inhibition of ovulation[b]
• Intrauterine device (IUD)
• Intrauterine hormone-releasing system (IUS)
• Bilateral tubal occlusion
• Vasectomized partner<br>*(Vasectomized partner is a highly effective contraceptive method provided that the partner is the sole sexual partner of the woman of childbearing potential and the absence of sperm has been confirmed. If not, additional highly effective method of contraception should be used. Spermatogenesis cycle is approximately 74 days.)*
**USER DEPENDENT**<br>**Highly Effective Methods That Are User Dependent** *Failure rate of <1% per year when used consistently and correctly*
• Combined (estrogen- and progestogen-containing) hormonal contraception associated with inhibition of ovulation[b]<br>– oral<br>– intravaginal<br>– transdermal<br>– injectable
• Progestogen-only hormone contraception associated with inhibition of ovulation[b]<br>– oral<br>– injectable
• Sexual abstinence<br>*(Sexual abstinence is considered a highly effective method only if defined as refraining from heterosexual intercourse during the entire period of risk associated with the study drug. The reliability of sexual abstinence needs to be evaluated in relation to the duration of the study and the preferred and usual lifestyle of the participant.)*
**NOT ALLOWED AS SOLE METHOD OF CONTRACEPTION DURING THE STUDY (not considered to be highly effective - failure rate of >1% per year)**
• Progestogen-only oral hormonal contraception where inhibition of ovulation is not the primary mode of action
• Male or female condom with or without spermicide[c]
• Cap, diaphragm, or sponge with spermicide
• A combination of male condom with either cap, diaphragm, or sponge with spermicide (double-barrier methods)[c]

• Periodic abstinence (calendar, symptothermal, post-ovulation methods)
• Withdrawal (coitus-interruptus)
• Spermicides alone
• Lactational amenorrhea method (LAM)
a) Typical use failure rates may differ from those when used consistently and correctly. Use should be consistent with local regulations regarding the use of contraceptive methods for participants in clinical studies.<br>b) Hormonal contraception may be susceptible to interaction with the study drug, which may reduce the efficacy of the contraceptive method. In addition, consider if the hormonal contraception may interact with the study drug.<br>c) Male condom and female condom should not be used together (due to risk of failure with friction).

Pregnancy During the Study

All initial reports of pregnancy in female participants or partners of male participants must be reported to the sponsor or designee by the study-site personnel within 24 hours of their knowledge of the event using the appropriate pregnancy notification form. Abnormal pregnancy outcomes (eg, spontaneous abortion, fetal death, stillbirth, congenital anomalies, ectopic pregnancy) are considered serious adverse events and must be reported using the Serious Adverse Event Form. Any participant who becomes pregnant during the study must be withdrawn study.

Follow-up information regarding the outcome of the pregnancy and any postnatal sequelae in the infant will be required.

10.4. Appendix 4: Regulatory, Ethical, and Study Oversight Considerations

REGULATORY AND ETHICAL CONSIDERATIONS

Investigator Responsibilities

The investigator is responsible for ensuring that the study is performed in accordance with the protocol, current International Conference on Harmonisation (ICH) guidelines on Good Clinical Practice (GCP), and applicable regulatory and country-specific requirements.

Good Clinical Practice is an international ethical and scientific quality standard for designing, conducting, recording, and reporting studies that involve the participation of human participants. Compliance with this standard provides public assurance that the rights, safety, and well-being of study participants are protected, consistent with the principles that originated in the Declaration of Helsinki, and that the study data are credible.

Protocol Amendments

Neither the investigator nor the sponsor will modify this protocol without a formal amendment by the sponsor. All protocol amendments must be issued by the sponsor, and signed and dated by the investigator. Protocol amendments must not be implemented without prior Independent Ethics Committee (IEC)/Institutional Review Board (IRB) approval, or when the relevant competent authority has raised any grounds for non-acceptance, except when necessary to eliminate immediate hazards to the participants, in which case the amendment must be promptly submitted to the IEC/IRB and relevant competent authority. Documentation of amendment approval by the investigator and IEC/IRB must be provided to the sponsor. When the change(s) involve only logistic or administrative aspects of the study, the IEC/IRB (where required) only needs to be notified.

During the course of the study, in situations where a departure from the protocol is unavoidable, the investigator or other physician in attendance will contact the appropriate sponsor representative listed in the Contact Information page(s), which will be provided as a separate document. Except in emergency situations, this contact should be made before implementing any departure from the protocol. In all cases, contact with the sponsor must be made as soon as possible to discuss the situation and agree on an appropriate course of action. The data recorded in the CRF and source documents will reflect any departure from the protocol, and the source documents will describe this departure and the circumstances requiring it.

Regulatory Approval/Notification

This protocol and any amendment(s) must be submitted to the appropriate regulatory authorities in each respective country, if applicable. A study may not be initiated until all local regulatory requirements are met.

Required Prestudy Documentation

The following documents must be provided to the sponsor before enrollment of the first participant

- Protocol and amendment(s), if any, signed and dated by the principal investigator
- A copy of the dated and signed (or sealed, where appropriate per local regulations), written IEC/IRB approval of the protocol, amendments, ICF, any recruiting materials, and if applicable, participant compensation programs. This approval must clearly identify the specific protocol by title and number and must be signed (or sealed, where appropriate per local regulations) by the chairman or authorized designee.
- Name and address of the IEC/IRB, including a current list of the IEC/IRB members and their function, with a statement that it is organized and operates according to GCP and the applicable laws and regulations. If accompanied by a letter of explanation, or equivalent, from the IEC/IRB, a general statement may be substituted for this list. If an investigator or a member of the study-site personnel is a member of the IEC/IRB, documentation must be obtained to state that this person did not participate in the deliberations or in the vote/opinion of the study.
- Regulatory authority approval or notification, if applicable
- Signed and dated statement of investigator (eg, Form FDA 1572), if applicable
- Documentation of investigator qualifications (eg, curriculum vitae)
- Completed investigator financial disclosure form from the principal investigator, where required
- Signed and dated clinical trial agreement, which includes the financial agreement
- Any other documentation required by local regulations

The following documents must be provided to the sponsor before enrollment of the first participant:

- Completed investigator financial disclosure forms from all subinvestigators
- Documentation of subinvestigator qualifications (eg, curriculum vitae)
- Name and address of any local laboratory conducting tests for the study, and a dated copy of current laboratory normal ranges for these tests, if applicable
- Local laboratory documentation demonstrating competence and test reliability (eg, accreditation/license), if applicable

Independent Ethics Committee or Institutional Review Board

Before the start of the study, the investigator (or sponsor where required) will provide the IEC/IRB with current and complete copies of the following documents (as required by local regulations):

- Final protocol and, if applicable, amendments
- Sponsor-approved ICF (and any other written materials to be provided to the participants)
- IB (or equivalent information) and amendments/addenda
- Sponsor-approved participant recruiting materials
- Information on compensation for study-related injuries or payment to participants for participation in the study, if applicable

- Investigator's curriculum vitae or equivalent information (unless not required, as documented by the IEC/IRB)
- Information regarding funding, name of the sponsor, institutional affiliations, other potential conflicts of interest, and incentives for participants
- Any other documents that the IEC/IRB requests to fulfill its obligation

This study will be undertaken only after the IEC/IRB has given full approval of the final protocol, amendments (if any, excluding the ones that are purely administrative, with no consequences for participants, data or study conduct, unless required locally), the ICF, applicable recruiting materials, and participant compensation programs, and the sponsor has received a copy of this approval. This approval letter must be dated and must clearly identify the IEC/IRB and the documents being approved.

During the study the investigator (or sponsor where required) will send the following documents and updates to the IEC/IRB for their review and approval, where appropriate:

- Protocol amendments (excluding the ones that are purely administrative, with no consequences for participants, data or study conduct)
- Revision(s) to ICF and any other written materials to be provided to participants
- If applicable, new or revised participant recruiting materials approved by the sponsor
- Revisions to compensation for study-related injuries or payment to participants for participation in the study, if applicable
- New edition(s) of the IB and amendments/addenda
- Summaries of the status of the study at intervals stipulated in guidelines of the IEC/IRB (at least annually)
- Reports of adverse events that are serious, unlisted/unexpected, and associated with the study drug
- New information that may adversely affect the safety of the participants or the conduct of the study
- Deviations from or changes to the protocol to eliminate immediate hazards to the participants
- Report of deaths of participants under the investigator's care
- Notification if a new investigator is responsible for the study at the site
- Development Safety Update Report and Line Listings, where applicable
- Any other requirements of the IEC/IRB

For all protocol amendments (excluding the ones that are purely administrative, with no consequences for participants, data or study conduct), the amendment and applicable ICF revisions must be submitted promptly to the IEC/IRB for review and approval before implementation of the change(s).

At least once a year, the IEC/IRB will be asked to review and reapprove this study, where required.

At the end of the study, the investigator (or sponsor where required) will notify the IEC/IRB about the study completion.

Other Ethical Considerations

For study-specific ethical design considerations, refer to Section 4.2.1.

FINANCIAL DISCLOSURE

Investigators and subinvestigators will provide the sponsor with sufficient, accurate financial information in accordance with local regulations to allow the sponsor to submit complete and accurate financial certification or disclosure statements to the appropriate regulatory authorities. Investigators are responsible for providing information on financial interests during the course of the study and for 1 year after completion of the study.

Refer to Required Prestudy Documentation (above) for details on financial disclosure.

INFORMED CONSENT PROCESS

Each participant must give written consent according to local requirements after the nature of the study has been fully explained. The ICF(s) must be signed before performance of any study-related activity. The ICF(s) that is/are used must be approved by both the sponsor and by the reviewing IEC/IRB and be in a language that the participant can read and understand. The informed consent should be in accordance with principles that originated in the Declaration of Helsinki, current ICH and GCP guidelines, applicable regulatory requirements, and sponsor policy.

Before enrollment in the study, the investigator or an authorized member of the study-site personnel must explain to potential participants the aims, methods, reasonably anticipated benefits, and potential hazards of the study, and any discomfort participation in the study may entail. Participants will be informed that their participation is voluntary and that they may withdraw consent to participate at any time. They will be informed that choosing not to participate will not affect the care the participant will receive for the treatment of his or her disease. Participants will be told that alternative treatments are available if they refuse to take part and that such refusal will not prejudice future treatment. Finally, they will be told that the investigator will maintain a participant identification register for the purposes of long-term follow-up if needed and that their records may be accessed by health authorities and authorized sponsor personnel without violating the confidentiality of the participant, to the extent permitted by the applicable law(s) or regulations. By signing the ICF the participant is authorizing such access.

The participant will be given sufficient time to read the ICF and the opportunity to ask questions. After this explanation and before entry into the study, consent should be appropriately recorded by means of the participant's personally dated signature. After having obtained the consent, a copy of the ICF must be given to the participant.

A participant who is rescreened is not required to sign another ICF if the rescreening occurs within 30 days from the previous ICF signature date.

If the participant is unable to read or write, an impartial witness should be present for the entire informed consent process (which includes reading and explaining all written information) and should personally date and sign the ICF after the oral consent of the participant is obtained.

When prior consent of the participant is not possible, enrollment procedures should be described in the protocol with documented approval/favorable opinion by the IEC/IRB to protect the rights, safety, and well-being of the participant and to ensure compliance with applicable regulatory requirements. The participant must be informed about the study as soon as possible and give consent to continue.

DATA PROTECTION

Privacy of Personal Data

The collection and processing of personal data from participants enrolled in this study will be limited to those data that are necessary to fulfill the objectives of the study.

These data must be collected and processed with adequate precautions to ensure confidentiality and compliance with applicable data privacy protection laws and regulations. Appropriate technical and organizational measures to protect the personal data against unauthorized disclosures or access, accidental or unlawful destruction, or accidental loss or alteration must be put in place. Sponsor personnel whose responsibilities require access to personal data agree to keep the identity of participants confidential.

The informed consent obtained from the participant includes explicit consent for the processing of personal data and for the investigator/institution to allow direct access to his or her original medical records (source data/documents) for study-related monitoring, audit, IEC/IRB review, and regulatory inspection. This consent also addresses the transfer of the data to other entities and to other countries.

The participant has the right to request through the investigator access to his or her personal data and the right to request rectification of any data that are not correct or complete. Reasonable steps will be taken to respond to such a request, taking into consideration the nature of the request, the conditions of the study, and the applicable laws and regulations.

LONG-TERM RETENTION OF SAMPLES FOR ADDITIONAL FUTURE RESEARCH

Not applicable

COMMITTEES STRUCTURE

Not applicable

[redacted]

[redacted]

The investigator understands that the information developed in the study will be used by the sponsor in connection with the continued development of itraconazole, and thus may be disclosed as required to other clinical investigators or regulatory agencies. To permit the information derived from the clinical studies to be used, the investigator is obligated to provide the sponsor with all data obtained in the study.

The results of the study will be reported in a Clinical Study Report generated by the sponsor and will contain data from all study sites that participated in the study as per protocol. Recruitment performance or specific expertise related to the nature and the key assessment parameters of the study will be used to determine a coordinating investigator for the study. Results of analyses performed after the Clinical Study Report has been issued will be reported in a separate report and will not require a revision of the Clinical Study Report.

Study participant identifiers will not be used in publication of results. Any work created in connection with performance of the study and contained in the data that can benefit from copyright protection (except any publication by the investigator as provided for below) shall be the property of the sponsor as author and owner of copyright in such work.

Consistent with Good Publication Practices and International Committee of Medical Journal Editors (ICMJE) guidelines, the sponsor shall have the right to publish such primary (multicenter) data and information without approval from the investigator. The investigator has the right to publish study site-specific data after the primary data are published. If an investigator wishes to publish information from the study, a copy of the manuscript must be provided to the sponsor for review at least 60 days before submission for publication or presentation. Expedited reviews will be arranged for abstracts, poster presentations, or other materials. If requested by the sponsor in writing, the investigator will withhold such publication for up to an additional 60 days to allow for filing of a patent application. In the event that issues arise regarding scientific integrity or regulatory compliance, the sponsor will review these issues with the investigator. The sponsor will not mandate modifications to scientific content and does not have the right to suppress information. For multicenter study designs and substudy approaches, secondary results generally should not be published before the primary endpoints of a study have been published. Similarly, investigators will recognize the integrity of a multicenter study by not submitting for publication data derived from the individual study site until the combined results from the completed study have been submitted for publication, within 18 months after the study end date, or the sponsor confirms there will be no multicenter study publication. Authorship of publications resulting from this study will be based on the guidelines on authorship, such as those described in the ICMJE Recommendations for the Conduct, Reporting, Editing and Publication of Scholarly Work in Medical Journals, which state that the named authors must have made a significant contribution to the conception or design of the work; or the acquisition, analysis, or interpretation of the data for the work; and drafted the work or revised it critically for important intellectual content; and given final approval of the version to be published; and agreed to be accountable for all aspects of the work in ensuring that

questions related to the accuracy or integrity of any part of the work are appropriately investigated and resolved.

Registration of Clinical Studies and Disclosure of Results

The sponsor will register and disclose the existence of and the results of clinical studies as required by law.

DATA QUALITY ASSURANCE

Data Quality Assurance/Quality Control

Steps to be taken to ensure the accuracy and reliability of data include the selection of qualified investigators and appropriate study sites, review of protocol procedures with the investigator and study-site personnel before the study, and periodic monitoring visits by the sponsor into the sponsor's data base.

Guidelines for CRF completion will be provided and reviewed with study-site personnel before the start of the study.

The sponsor will review CRF for accuracy and completeness during on-site monitoring visits and after transmission to the sponsor; any discrepancies will be resolved with the investigator or designee, as appropriate. After upload of the data into the study database they will be verified for accuracy and consistency with the data sources.

CASE REPORT FORM COMPLETION

Case report forms are prepared and provided by the sponsor for each participant in electronic format. All data relating to the study must be recorded in CRF. All CRF entries, corrections, and alterations must be made by the investigator or authorized study-site personnel. The investigator must verify that all data entries in the CRF are accurate and correct.

The study data will be transcribed by study-site personnel from the source documents onto an electronic CRF, if applicable. Study-specific data will be transmitted in a secure manner to the sponsor.

Worksheets may be used for the capture of some data to facilitate completion of the CRF. Any such worksheets will become part of the participant's source documents. Data must be entered into CRF in English. The CRF must be completed as soon as possible after a participant visit and the forms should be available for review at the next scheduled monitoring visit.

If necessary, queries will be generated in the electronic data capture (eDC) tool. If corrections to a CRF are needed after the initial entry into the CRF, this can be done in either of the following ways:

- Investigator and study-site personnel can make corrections in the eDC tool at their own initiative or as a response to an auto query (generated by the eDC tool).

- Sponsor or sponsor delegate can generate a query for resolution by the investigator and study-site personnel.

SOURCE DOCUMENTS

At a minimum, source documents consistent in the type and level of detail with that commonly recorded at the study site as a basis for standard medical care must be available for the following: participant identification, eligibility, and study identification; study discussion and date of signed informed consent; dates of visits; results of safety and efficacy parameters as required by the protocol; record of all adverse events and follow-up of adverse events; concomitant medication; drug receipt/dispensing/return records; study drug administration information; and date of study completion and reason for early discontinuation of study drug or withdrawal from the study, if applicable.

The author of an entry in the source documents should be identifiable.

Specific details required as source data for the study and source data collection methods will be reviewed with the investigator before the study and will be described in the monitoring guidelines (or other equivalent document).

The following data will be recorded directly into the CRF and will be considered source data

- Race
- Sex
- History of smoking, all nicotine use, eg, cigarettes (including e-cigarettes or the equivalent of e-cigarettes), cigars, chewing tobacco, patch, and gum
- Blood pressure and pulse/heart rate
- Height and weight
- Details of physical examination
- Investigator Global Evaluation tool assessments

An eSource system may be utilized, which contains data traditionally maintained in a hospital or clinic record to document medical care (eg, electronic source documents) as well as the clinical study-specific data fields as determined by the protocol. This data is electronically extracted for use by the sponsor. If eSource is utilized, references made to the CRF in the protocol include the eSource system but information collected through eSource may not be limited to that found in the CRF.

MONITORING

The sponsor will use a combination of monitoring techniques on-site monitoring to monitor this study.

The sponsor will perform on-site monitoring visits as frequently as necessary. The monitor will record dates of the visits in a study-site visit log that will be kept at the study site. The first post-

initiation visit will be made as soon as possible after enrollment has begun. At these visits, the monitor will compare the data entered into the CRF with the source documents (eg, hospital/clinic/physician's office medical records). The nature and location of all source documents will be identified to ensure that all sources of original data required to complete the CRF are known to the sponsor and study-site personnel and are accessible for verification by the sponsor study-site contact. If electronic records are maintained at the study site, the method of verification must be discussed with the study-site personnel.

Direct access to source documents (medical records) must be allowed for the purpose of verifying that the recorded data are consistent with the original source data. Findings from this review will be discussed with the study-site personnel. The sponsor expects that, during monitoring visits, the relevant study-site personnel will be available, the source documents will be accessible, and a suitable environment will be provided for review of study-related documents. The monitor will meet with the investigator on a regular basis during the study to provide feedback on the study conduct.

ON-SITE AUDITS

Representatives of the sponsor's clinical quality assurance department may visit the study site at any time during or after completion of the study to conduct an audit of the study in compliance with regulatory guidelines and company policy. These audits will require access to all study records, including source documents, for inspection. Participant privacy must, however, be respected. The investigator and study-site personnel are responsible for being present and available for consultation during routinely scheduled study-site audit visits conducted by the sponsor or its designees.

Similar auditing procedures may also be conducted by agents of any regulatory body, either as part of a national GCP compliance program or to review the results of this study in support of a regulatory submission. The investigator should immediately notify the sponsor if he or she has been contacted by a regulatory agency concerning an upcoming inspection.

RECORD RETENTION

In compliance with the ICH/GCP guidelines, the investigator/institution will maintain all CRF and all source documents that support the data collected from each participant, as well as all study documents as specified in ICH/GCP Section 8, Essential Documents for the Conduct of a Clinical Trial, and all study documents as specified by the applicable regulatory requirement(s). The investigator/institution will take measures to prevent accidental or premature destruction of these documents.

Essential documents must be retained until at least 2 years after the last approval of a marketing application in an ICH region and until there are no pending or contemplated marketing applications in an ICH region or until at least 2 years have elapsed since the formal discontinuation of clinical development of the investigational product. These documents will be retained for a longer period if required by the applicable regulatory requirements or by an agreement with the sponsor. It is the

responsibility of the sponsor to inform the investigator/institution as to when these documents no longer need to be retained.

If the responsible investigator retires, relocates, or for other reasons withdraws from the responsibility of keeping the study records, custody must be transferred to a person who will accept the responsibility. The sponsor must be notified in writing of the name and address of the new custodian. Under no circumstance shall the investigator relocate or dispose of any study documents before having obtained written approval from the sponsor.

If it becomes necessary for the sponsor or the appropriate regulatory authority to review any documentation relating to this study, the investigator/institution must permit access to such reports.

STUDY AND SITE CLOSURE

Study Termination

The sponsor reserves the right to close the study site or terminate the study at any time for any reason at the sole discretion of the sponsor. Study sites will be closed upon study completion. A study site is considered closed when all required documents and study supplies have been collected and a study-site closure visit has been performed.

The investigator may initiate study-site closure at any time, provided there is reasonable cause and sufficient notice is given in advance of the intended termination.

Reasons for the early closure of a study site by the sponsor or investigator may include but are not limited to:

- Failure of the investigator to comply with the protocol, the requirements of the IEC/IRB or local health authorities, the sponsor's procedures, or GCP guidelines
- Inadequate recruitment of participants by the investigator
- Discontinuation of further study drug development

10.5. Appendix 5: Adverse Events: Definitions and Procedures for Recording, Evaluating, Follow-up, and Reporting

ADVERSE EVENT DEFINITIONS AND CLASSIFICATIONS

Adverse Event

An adverse event is any untoward medical occurrence in a clinical study participant administered a medicinal (investigational or non-investigational) product. An adverse event does not necessarily have a causal relationship with the drug. An adverse event can therefore be any unfavorable and unintended sign (including an abnormal finding), symptom, or disease temporally associated with the use of a medicinal (investigational or non-investigational) product, whether or not related to that medicinal (investigational or non-investigational) product (Definition per ICH).

This includes any occurrence that is new in onset or aggravated in severity or frequency from the baseline condition, or abnormal results of diagnostic procedures, including laboratory test abnormalities.

Note: The sponsor collects adverse events starting with the signing of the ICF (refer to All Adverse Events under Section 8.3.1, Time Period and Frequency for Collecting Adverse Events and Serious Adverse Events Information, for time of last adverse event recording).

Serious Adverse Event

A serious adverse event based on ICH and EU Guidelines on Pharmacovigilance for Medicinal Products for Human Use is any untoward medical occurrence that at any dose:

- Results in death
- Is life-threatening
  (The participant was at risk of death at the time of the event. It does not refer to an event that hypothetically might have caused death if it were more severe).
- Requires inpatient hospitalization or prolongation of existing hospitalization
- Results in persistent or significant disability/incapacity
- Is a congenital anomaly/birth defect
- Is a suspected transmission of any infectious agent via a medicinal product
- Is Medically Important*

*Medical and scientific judgment should be exercised in deciding whether expedited reporting is also appropriate in other situations, such as important medical events that may not be immediately life threatening or result in death or hospitalization but may jeopardize the participant or may require intervention to prevent one of the other outcomes listed in the definition above. These should usually be considered serious.

If a serious and unexpected adverse event occurs for which there is evidence suggesting a causal relationship between the study drug and the event (eg, death from anaphylaxis), the event must be

reported as a serious and unexpected suspected adverse reaction even if it is a component of the study endpoint (eg, all-cause mortality).

Unlisted (Unexpected) Adverse Event/Reference Safety Information

An adverse event is considered unlisted if the nature or severity is not consistent with the applicable product reference safety information. For itraconazole, the expectedness of an adverse event will be determined by whether or not it is listed in Section 4.8 (Undesirable effects) of the SmPC.[20]

Adverse Event Associated With the Use of the Intervention

An adverse event is considered associated with the use of the drug if the attribution is possible, probable, or very likely by the definitions listed below (see Attribution Definitions).

ATTRIBUTION DEFINITIONS

Not Related

An adverse event that is not related to the use of the drug.

Doubtful

An adverse event for which an alternative explanation is more likely, eg, concomitant treatment(s), concomitant disease(s), or the relationship in time suggests that a causal relationship is unlikely.

Possible

An adverse event that might be due to the use of the drug. An alternative explanation, eg, concomitant treatment(s), concomitant disease(s), is inconclusive. The relationship in time is reasonable; therefore, the causal relationship cannot be excluded.

Probable

An adverse event that might be due to the use of the drug. The relationship in time is suggestive (eg, confirmed by dechallenge). An alternative explanation is less likely, eg, concomitant treatment(s), concomitant disease(s).

Very Likely

An adverse event that is listed as a possible adverse reaction and cannot be reasonably explained by an alternative explanation, eg, concomitant treatment(s), concomitant disease(s). The relationship in time is very suggestive (eg, it is confirmed by dechallenge and rechallenge).

SEVERITY CRITERIA

An assessment of severity grade will be made using the following general categorical descriptors:

Mild: Awareness of symptoms that are easily tolerated, causing minimal discomfort and not interfering with everyday activities.

Moderate: Sufficient discomfort is present to cause interference with normal activity.

Severe: Extreme distress, causing significant impairment of functioning or incapacitation. Prevents normal everyday activities.

The investigator should use clinical judgment in assessing the severity of events not directly experienced by the participant (eg, laboratory abnormalities).

SPECIAL REPORTING SITUATIONS

Safety events of interest on a sponsor study drug in an interventional study that may require expedited reporting or safety evaluation include, but are not limited to:

- Overdose of a sponsor study drug
- Suspected abuse/misuse of a sponsor study drug
- Accidental or occupational exposure to a sponsor study drug
- Any failure of expected pharmacologic action (ie, lack of effect) of a sponsor study drug
- Unexpected therapeutic or clinical benefit from use of a sponsor study drug
- Medication error involving a sponsor product (with or without participant/patient exposure to the sponsor study drug, eg, name confusion)
- Exposure to a sponsor study drug from breastfeeding

Special reporting situations should be recorded in the CRF. Any special reporting situation that meets the criteria of a serious adverse event should be recorded on the serious adverse event page of the CRF.

PROCEDURES

All Adverse Events

All adverse events, regardless of seriousness, severity, or presumed relationship to study drug, must be recorded using medical terminology in the source document and the CRF. Whenever possible, diagnoses should be given when signs and symptoms are due to a common etiology (eg, cough, runny nose, sneezing, sore throat, and head congestion should be reported as "upper respiratory infection"). Investigators must record in the CRF their opinion concerning the relationship of the adverse event to study therapy. All measures required for adverse event management must be recorded in the source document and reported according to sponsor instructions.

For all studies with an outpatient phase, including open-label studies, the participant must be provided with a "wallet (study) card" and instructed to carry this card with them for the duration of the study indicating the following:

- Study number
- Statement, in the local language(s), that the participant is participating in a clinical study
- Investigator's name and 24-hour contact telephone number

- Local sponsor's name and 24-hour contact telephone number (for medical staff only)
- Site number
- Participant number
- Any other information that is required to do an emergency breaking of the blind

Serious Adverse Events

All serious adverse events that have not resolved by the end of the study, or that have not resolved upon discontinuation of the participant's participation in the study, must be followed until any of the following occurs:

- The event resolves
- The event stabilizes
- The event returns to baseline, if a baseline value/status is available
- The event can be attributed to agents other than the study drug or to factors unrelated to study conduct
- It becomes unlikely that any additional information can be obtained (participant or health care practitioner refusal to provide additional information, lost to follow-up after demonstration of due diligence with follow-up efforts)

Suspected transmission of an infectious agent by a medicinal product will be reported as a serious adverse event. Any event requiring hospitalization (or prolongation of hospitalization) that occurs during the course of a participant's participation in a study must be reported as a serious adverse event, except hospitalizations for the following:

- Hospitalizations not intended to treat an acute illness or adverse event (eg, social reasons such as pending placement in long-term care facility)
- Surgery or procedure planned before entry into the study (must be documented in the CRF). Note: Hospitalizations that were planned before the signing of the ICF, and where the underlying condition for which the hospitalization was planned has not worsened, will not be considered serious adverse events. Any adverse event that results in a prolongation of the originally planned hospitalization is to be reported as a new serious adverse event.
- For convenience the investigator may choose to hospitalize the participant for the duration of the intervention period.

The cause of death of a participant in a study within 30 days of the last dose of study drug, whether or not the event is expected or associated with the study drug, is considered a serious adverse event.

CONTACTING SPONSOR REGARDING SAFETY

The names (and corresponding telephone numbers) of the individuals who should be contacted regarding safety issues or questions regarding the study are listed in the Contact Information page(s), which will be provided as a separate document.

PRODUCT QUALITY COMPLAINT HANDLING

A product quality complaint (PQC) is defined as any suspicion of a product defect related to manufacturing, labeling, or packaging, ie, any dissatisfaction relative to the identity, quality, durability, or reliability of a product, including its labeling or package integrity. A PQC may have an impact on the safety and efficacy of the product. Timely, accurate, and complete reporting and analysis of PQC information from studies are crucial for the protection of participants, investigators, and the sponsor, and are mandated by regulatory agencies worldwide. The sponsor has established procedures in conformity with regulatory requirements worldwide to ensure appropriate reporting of PQC information; all studies conducted by the sponsor or its affiliates will be conducted in accordance with those procedures.

Procedures

All initial PQCs must be reported to the sponsor by the study-site personnel within 24 hours after being made aware of the event.

If the defect is combined with a serious adverse event, the study-site personnel must report the PQC to the sponsor according to the serious adverse event reporting timelines (refer to Section 8.3.1, Time Period and Frequency for Collecting Adverse Event and Serious Adverse Event Information). A sample of the suspected product should be maintained for further investigation if requested by the sponsor.

Contacting Sponsor Regarding Product Quality

The names (and corresponding telephone numbers) of the individuals who should be contacted regarding product quality issues are listed in the Contact Information page(s), which will be provided as a separate document.

10.6. Appendix 6: Clinical Laboratory Tests

The following tests will be performed according to the SoA by the local and central laboratory:

Table 3: Protocol-Required Safety Laboratory Assessments

Laboratory Assessments	Parameters
Screening Tests	• Serum pregnancy testing for women of childbearing potential and not on any effective contraceptive measure only

Table 4: Protocol-Required Laboratory Assessments

Laboratory Assessments	Parameters
Blood Test	• Plasma drug concentration of itraconazole and hydroxy-itraconazole of all participants completing treatment
Mycological Test	• KOH mount for all participants at baseline to confirm fungal infection and at end of study to assess mycological cure<br>• Culture and sensitivity for all participants at baseline to confirm type of fungi and its sensitivity to oral antifungal drugs and at end of study to assess mycological cure

10.7. Appendix 7: Protocol Amendment History

**DOCUMENT HISTORY**	
**Document**	**Date**
Amendment 1	08-Feb-2019
Original Protocol	28-Jun-2018

11. REFERENCES

1. American Red Cross. http://www.redcrossblood.org/learn-about-blood/blood-facts-and-statistics. Accessed 16 March 2018.
2. Clinical Research Report ITR-INT-47. Effects of itraconazole in the treatment of superficial dermatomycoses of the glabrous skin (tinea corporis/cruris, Candida intertrigo). A randomized, double-blind comparison with terbinafine. Janssen Research Foundation (August 1995).
3. Clinical Research Report ITR-NED-6. Itraconazole in tinea corporis and tinea cruris: comparison of two treatment schedules. Janssen Research Foundation (October 1995).
4. Clinical Study Report ITR-INT-18. Randomised, Double-Blind Trial of the Efficacy and Tolerability of Itraconazole 200mg Compared With Terbinafine 250mg in the Treatment of Tinea corporis/cruris. Janssen Research Foundation (October 1995)
5. Cohn, M.S. Superficial fungal infections: topical and oral treatment of common types. Postgrad Med. 1992;91(2):239-252.
6. Company Core Data Sheet (CCDS). Itraconazole Capsules. Janssen Research & Development, LLC. (16 August 2017).
7. Dogra S, Uprety S. The menace of chronic and recurrent dermatophytosis in India: Is the problem deeper than we perceive? Indian Dermatol Online J. 2016;7:73-76.
8. Investigator's Brochure. Itraconazole capsules R051211. Fifth Edition. Janssen (May 1986).
9. Investigator's Brochure. Itraconazole tablets R051211. Edition 1. Janssen (March 1999).
10. Johnson MLT. Skin conditions and related need for medical care among persons 1-74 years, United States, 1971-1972. Dept. HEW, series 11, number 212, DHEW Publication number (PHS), 79-1660 (November 1978).
11. Jones HE. Incidence and importance of candidiasis in dermatomycosis - treatment with ketoconazole. In Meinhof, W.,ed. Oral therapy in dermatomycosis: a step forward. Medicine Publishing Foundation, Symposium series, Oxford, Medical Education Services, 7-18 (1985).
12. Mac Donald E, Smith EB. The geography of the dermatophytes. Dermatologic clinics: 85-91, Philadelphia, 1984.
13. Majid I, Sheikh G, Kanth F, Hakak R. Relapse after oral terbinafine therapy in dermatophytosis: A clinical and mycological study. Indian J Dermatol. 2016;61:529-533.
14. Odom R. Pathophysiology of dermatophyte infection. J Am Acad Dermatol. 1993;28:S2-S7.
15. Panda S, Verma S. The menace of dermatophytosis in India: The evidence that we need. Indian J Dermatol Venereol Leprol. 2017;83(3):281-284.
16. Periodic Benefit Risk Evaluation Report/Periodic Safety Update Report (PBRER/PSUR). Itraconazole and Itraconazole/Secnidazole. Janssen Research & Development, LLC, (17 May 2017).
17. Seebacker C, Blaschke-Hellmessen R. Mykosen, epidemiologie-diagnostik-therapie. Gustav Fisher Verl Jena. 1990; 279:40-46.
18. Sinski JT, Kelley LM. A survey of dermatophytes from human patients in the United States from 1985 to 1987. Mycopathologia. 1991;114:117-126.
19. Clinical Expert Report. Itraconazole 100 mg capsules for dermatomycosis. Summary of the efficacy and safety of a treatment of 1 week with 200 mg o.d. for tinea corporis/cruris and 200 mg b.i.d. for plantar tinea pedis. Janssen Research Foundation (February 1996).
20. Sporanox Capsule Summary of Product Characteristics. Buckinghamshire, UK. Janssen-Cilag Ltd; 2013.
21. Sporanox package insert. Mumbai, India. Janssen Pharmaceuticals, Inc; 2017.
22. Stem RS. The epidemiology of cutaneous disease. Fitzpatrick TB, Eisen AZ, Wolff K, Freedberg IM, Austen KF, eds. Dermatology in general Medicine, New York NY: Mc Graw Hill International Book Co.; 1993:7-13.

23. Trial identification and protocol summary ITR-BEL-2. Clinical experience with short schedules of itraconazole in the treatment of tinea corporis and/or tinea cruris. Janssen Research Foundation (October 1995).

24. Tripathy S. Rising prevalence of dermatophytosis in India: A matter of concern. Int J of Infect Dis. 2016;45:318.

25. Verma S, Madhu R. The great Indian epidemic of superficial dermatophytosis: An appraisal. Indian J Dermatol. 2017;62:227-236.

INVESTIGATOR AGREEMENT

I have read this protocol and agree that it contains all necessary details for carrying out this study. I will conduct the study as outlined herein and will complete the study within the time designated.

I will provide copies of the protocol and all pertinent information to all individuals responsible to me who assist in the conduct of this study. I will discuss this material with them to ensure that they are fully informed regarding the study drug, the conduct of the study, and the obligations of confidentiality.

Coordinating Investigator (where required):

Name (typed or printed): ____________________

Institution and Address: ____________________

____

____

____

Signature: ____________________ Date: ____________________

(Day Month Year)

Principal (Site) Investigator:

Name (typed or printed): ____________________

Institution and Address: ____________________

____

____

____

Telephone Number: ____________________

Signature: ____________________ Date: ____________________

(Day Month Year)

Sponsor's Responsible Medical Officer:

Name (typed or printed): PPD, MD

Institution: Janssen Research & Development , LLC

Signature: electronic signature appended at the end of the protocol Date: ____________________

(Day Month Year)

Note: If the address or telephone number of the investigator changes during the course of the study, written notification will be provided by the investigator to the sponsor, and a protocol amendment will not be required.
