# Supplementary material for: No evidence of resistance to itraconazole in a prospective real-world trial of dermatomycosis in India
Source: PLoS One. 2023 Feb 14;18(2):e0281514. doi: 10.1371/journal.pone.0281514 (PMC9928099; doi:10.1371/journal.pone.0281514)
Supplement: S3 File — (PDF) [file pone.0281514.s004.pdf]

Janssen Research & Development

Statistical Analysis Plan

Itraconazole in the Management of Superficial Fungal Infections in India: A Pilot Study.

Protocol R051211FUN4058; Phase 4

JNJ-16269994 (itraconazole)

**Status:**	Approved
**Date:**	5 June 2020
**Prepared by:**	Janssen Research & Development, LLC
**Document No.:**	EDMS-EDMS-ERI-160717658, 1.0

Compliance: The study described in this report was performed according to the principles of Good Clinical Practice (GCP).

Confidentiality Statement

The information in this document contains trade secrets and commercial information that are privileged or confidential and may not be disclosed unless such disclosure is required by applicable law or regulations. In any event, persons to whom the information is disclosed must be informed that the information is privileged or confidential and may not be further disclosed by them. These restrictions on disclosure will apply equally to all future information supplied to you that is indicated as privileged or confidential.

SIGNATURE PAGE

Author: ____
TCS Statistician

Reviewer: ____
JRD Statistician

JRD Study Responsible Physician: ____________________________
JRD SRP

TABLE OF CONTENTS

TABLE OF CONTENTS 3

AMENDMENT HISTORY 4

ABBREVIATIONS 5

1. INTRODUCTION 6
1.1. Trial Objectives 6
1.2. Trial Design 6
1.3. Statistical Hypotheses for Trial Objectives 8
1.4. Sample Size Justification 8
1.5. Randomization and Blinding 8

2. GENERAL ANALYSIS DEFINITIONS 8
2.1. Analysis Sets 8
2.1.1. All Enrolled Analysis Set 8
2.1.2. Efficacy Analysis Set (intent-to-treat population [ITT]) 8
2.1.3. Safety Analysis Set 8
2.1.4. Pharmacokinetics Analysis Set 8
2.1.5. Completer Analysis Set 9
2.2. Study Day and Relative Day 9
2.3. Baseline and Endpoint 9
2.4. General Analysis Rules 9

3. INTERIM ANALYSIS AND DATA MONITORING COMMITTEE REVIEW 9

4. SUBJECT INFORMATION 9
4.1. Demographics and Baseline Characteristics 10
4.2. Disposition Information 10
4.3. Treatment Compliance 11
4.4. Extent of Exposure 11
4.5. Protocol Deviations 11
4.6. Prior and Concomitant Medications 11
4.7. Medical history 11

5. EFFICACY 12
5.1. Analysis Specifications 12
5.1.1. Level of Significance 12
5.2. Primary Efficacy Endpoint 12
5.2.1. Definition 12
5.2.2. Analysis Methods 12
5.3. Major Secondary Endpoints 12
5.3.1. Definition 13
5.3.2. Analysis Methods 13

6. SAFETY 14
6.1. Adverse Events 14
6.2. Clinical Laboratory Tests and Vital Signs Findings 14

7. PHARMACOKINETICS/PHARMACODYNAMICS 15
7.1. Pharmacokinetics 15

8. BIOMARKERS 15

9. HEALTH ECONOMICS 15

REFERENCES 16

AMENDMENT HISTORY

Not applicable

ABBREVIATIONS

AE	adverse event
ATC	anatomic and therapeutic Class
BMI	body mass index
BSA	body surface area
CI	confidence interval
CRF	case report form
CSR	Clinical Study Report
CV	coefficient of variation
DMC	Data Monitoring Committee
DPS	Data Presentation Specifications
ECG	electrocardiogram
eCRF	electronic case report form
FAS	full analysis set
FDA	Food and Drug Administration
ICH	International Conference on Harmonization
IQ	interquartile
MedDRA	Medical Dictionary for Regulatory Activities
PD	pharmacodynamic
PK	pharmacokinetic(s)
SAE	serious adverse event
SAP	Statistical Analysis Plan
SD	standard deviation
SMQs	standardised MedDRA queries
TEAE	treatment-emergent adverse event
WHO	World Health Organization
WHO-DD	World Health Organization Drug Dictionary
ITT	Intention-to-Treat

1. INTRODUCTION

This study is designed to evaluate clinical outcomes and risk factors associated with clinical response in adult participants from India with T. cruris or T. corporis treated with itraconazole as part of their clinical care.

This statistical analysis plan (SAP) for the R051211FUN4058 describes the statistical analysis for subject information, safety, and efficacy data.

1.1. Trial Objectives

Primary Objective

- To estimate the proportion of participants prescribed itraconazole for T.cruris or T.corporis who have clinical response after 7 days of treatment.

Secondary Objective

- Estimate the proportion of participants prescribed itraconazole for T.cruris or T.corporis who have mycological response after 14 days of follow-up.
- Association of the clinical response at Day 7 with plasma drug concentrations of itraconazole and hydroxy-itraconazole.
- Association of the clinical response at Day 7 with the baseline sensitivity pattern of causative fungi.
- Estimate proportion of participants with clinical response after 14 days of follow-up. Estimate the extent to which clinically improved at Day 7 predicts clinically improved at Day 14.

Tertiary/Exploratory Objective

- Evaluate the proportion of participants that come for follow-up after 7 days of treatment.

1.2. Trial Design

This is a real-world, prospective, non-randomized, open-label, multicenter, interventional, longitudinal pilot study to evaluate clinical outcomes and risk factors associated with clinical response in adult participants from India with T.cruris or T.corporis treated with itraconazole, including reference itraconazole, as part their clinical care. 50 participants consisting of men and women between 18 to 60 years of age with T.cruris or T.corporis infection that have been prescribed itraconazole 200 mg daily will be enrolled in this study. The study will be conducted at 4 to 5 large clinics of dermatologists across different regions of India and will include sites that have their own pharmacies that dispense itraconazole and reference itraconazole. A minimum of 6 participants treated with reference itraconazole will be included or the mitigation plan of study extension will be implemented.

The study will consist of 3 phases: a screening phase; a 7-day open-label treatment phase; a 7-day observation phase; and end of study (EOS) visit at Day 14. The duration of individual participation will be approximately 15 days.

A diagram of the study design is provided below in Figure 1.

Figure 1: Schematic Overview of the Study

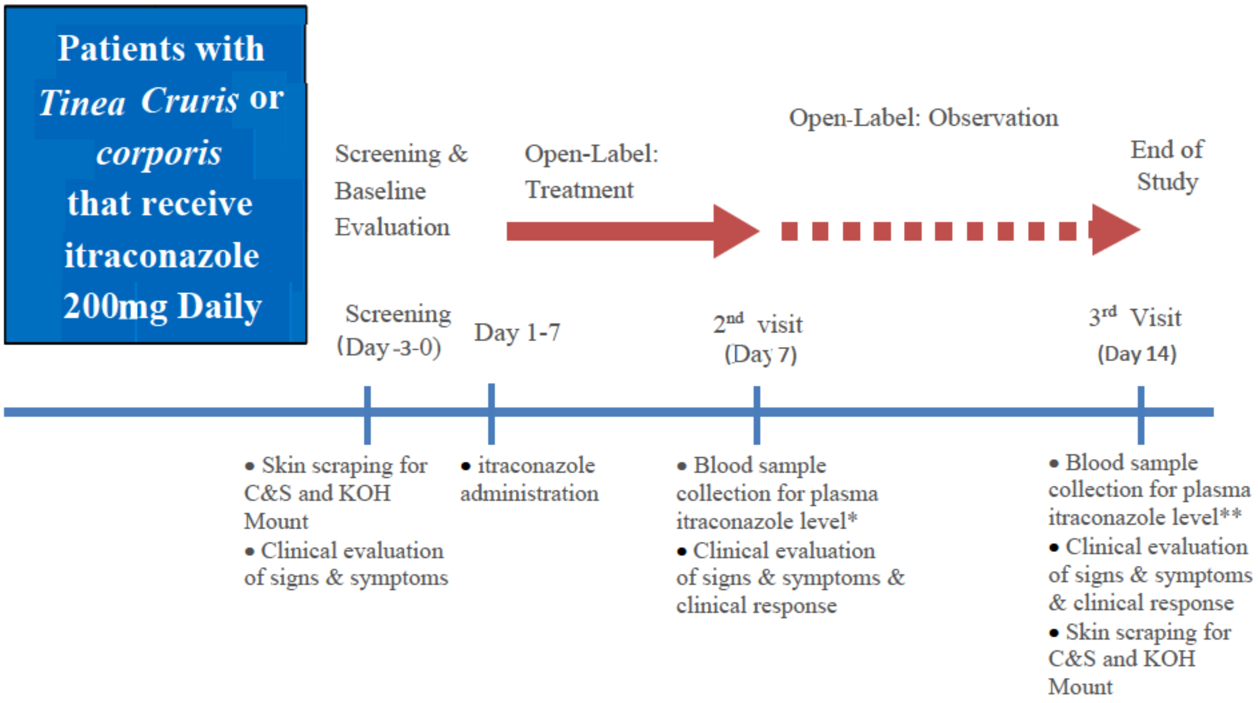

*Blood Samples Collection: 24±2 hours after previous dose (Day 6, Day 13) and 2 and 4.5 hours ±10 minutes after the last dose (Day 7, Day 14).
**Only in participants that continue receiving itraconazole at the discretion of the treating physician as standard practice.
Key: C&S=culture and itraconazole sensitivity; KOH=potassium hydroxide.

1.3. Statistical Hypotheses for Trial Objectives

The primary efficacy endpoint is defined as the estimated proportion of participants who have clinical response ("healed" or "markedly improved" of the Investigator Global Evaluation Tool of clinical improvement) after 7 days of treatment and hence no formal hypothesis testing will be conducted. The point estimate and the corresponding 95% Confidence Interval for the efficacy endpoints will be provided.

1.4. Sample Size Justification

This study is a pilot study and the sample size was not calculated based on power, rather it was selected to ensure a minimum number of participants with reference itraconazole, (at least 6) out of 50 participants total.

After the inclusion of 12 participants in the study the number of participants with reference itraconazole will be evaluated. If there are less than 3 participants receiving reference itraconazole a mitigation plan will be implemented with the inclusion of one more site with pharmacy to increase the number of participants treated with reference itraconazole.

1.5. Randomization and Blinding

As this is a non-randomized and open label study, randomization and blinding procedures are not applicable.

2. GENERAL ANALYSIS DEFINITIONS

2.1. Analysis Sets

2.1.1. All Enrolled Analysis Set

All enrolled analysis set is defined as those participants who have signed the informed consent, and are enrolled in the study.

2.1.2. Efficacy Analysis Set (intent-to-treat population [ITT])

Efficacy analysis set includes participants who have completed the 7-day treatment regime with prescribed itraconazole and have had clinical evaluations at baseline and 7 days.

2.1.3. Safety Analysis Set

The safety analysis set includes all participants that have received at least 1 administration of prescribed itraconazole.

2.1.4. Pharmacokinetics Analysis Set

The pharmacokinetics (PK) analysis set includes all participants that have at least one set of blood samples drawn at Day 7 to evaluate plasma drug concentrations of itraconazole and its main metabolite, hydroxy-itraconazole.

2.1.5. Completer Analysis Set

Completer analysis set includes participants who have completed the 7-day treatment regime and have had clinical evaluations at baseline, 7 days and at 14 days.

2.2. Study Day and Relative Day

Study Day 1 or Day 1 refers to the start of the first study agent administration. All efficacy and safety assessments at all visits will be assigned a day relative to this date.

Study day or relative day for a visit is defined as:

- Visit date - (date of Study Day 1) +1, if visit date is ≥date of Day 1
- Visit date - Date of Day 1, if visit date <date of Day 1

There is no 'Day 0'

2.3. Baseline and Endpoint

Baseline is defined as the last observation prior to the start of the first study agent administrations.

Endpoint is defined as the last available post-baseline result at Day 7 and Day 14 within the analysis period. Unscheduled visit results are included in this definition and will be considered as the endpoint value if the unscheduled visit result is the last post-baseline result available within the analysis period.

2.4. General Analysis Rules

- Continuous data will be summarized by descriptive statistics, including number of subjects (N), mean, standard deviation (SD), median, and range (minimum; maximum).
- The minimum and maximum will be presented to the same number of decimal places as the original data. The mean, median and 95% CI will be rounded to one additional decimal place than the original data, while SD will be approximated to two additional decimal places.
- If a count is 0, the percentage (0%) should not be displayed. The 0 count will be displayed, but the corresponding percentage should be omitted.
- The percentages (%) in tables will be presented to 1 decimal place unless the sample sizes for the percentages are small enough to warrant presenting as integers.

3. INTERIM ANALYSIS AND DATA MONITORING COMMITTEE REVIEW

There is no interim analysis planned for this study.

4. SUBJECT INFORMATION

Subject information will be tabulated by treatment.

4.1. Demographics and Baseline Characteristics

Table 01 presents a list of the demographic variables that will be summarized for all enrolled analysis set.

Table 01: Demographic Variables

**Continuous Variables**	**Summary Type**
Age (years)	Descriptive statistics (N, mean, standard deviation [SD], median and range [minimum and maximum])
Weight (kg)	
Height (cm)	
BMI (kg/m^2)	
Body surface (m^2)	
**Categorical Variables**	
Sex (male, female, Unknown, undifferentiated)	Frequency distribution with the number and percentage of subjects in each category.
Ethnicity (Indian, Non-Indian, Not Reported, Unknown)	
Childbearing potential(Of childbearing potential, Permanently Sterilized, Postmenopausal, Not Applicable)	

The demographics and baseline characteristics will be listed for all subjects.

Data on Substance use will be listed.

A listing of Comments will be provided.

Total scores of all signs and symptoms calculated on Clinical Evaluation Tool and KOH mount result (Positive, Negative) at screening will be summarized as baseline characteristics for all enrolled analysis set.

4.2. Disposition Information

The number of subjects in the following disposition categories will be summarized throughout the study by treatment:

- Subjects receiving study agent
- Subjects completing the study
- Subjects who discontinued study agent
- Reasons for discontinuation of study agent
- Subjects who terminated study prematurely
- Reasons for termination of study

Listings of subjects will be provided for the following categories:

- Subjects who discontinued study agent
- Subjects who terminated study prematurely

The number of subjects in the following disposition categories will be summarized:

- Adverse Event
- Death
- Lack of Efficacy
- Lost to Follow-Up
- Initiated Prohibited Medication
- Noncompliance as subject failed to take 80% of study drug
- Physician Decision
- Pregnancy
- Product Quality Complaint
- Study Terminated by Sponsor
- Subject Refused Further Study Treatment
- Withdrawal of Consent
- Anaphylactic reaction of study drug
- Other

4.3. Treatment Compliance

Not applicable for this study

4.4. Extent of Exposure

Not applicable for this study

4.5. Protocol Deviations

If captured, major protocol deviations will be defined. A listing of subjects with a major protocol deviation will be provided.

4.6. Prior and Concomitant Medications

Prior and Concomitant medications will be coded using the World Health Organization Drug Dictionary (WHO-DD).
Prior medications are defined as any therapy used before the day of first dose (partial or complete) of study agent. Concomitant medications are defined as any therapy used on or after the same day as the first dose of study agent, including those that started before and continue on after the first dose of study agent. If both start date and end date are missing, then this medication will be considered as both prior and concomitant medications.

Summaries of Prior and Concomitant medications will be presented by ATC term. The data on prior and concomitant medications will be provided in separate listings.

4.7. Medical history

Medical History will be listed for all subjects.

5. EFFICACY

5.1. Analysis Specifications

5.1.1. Level of Significance

All analyses will be descriptive. Although no hypothesis testing will be performed, the point estimate and the corresponding 95% Confidence Interval for the efficacy endpoints will be provided.

5.2. Primary Efficacy Endpoint

The primary efficacy endpoint is defined as the estimated proportion of participants who have clinical response ("healed" or "markedly improved" of the Investigator Global Evaluation Tool of clinical improvement) after 7 days of treatment.

5.2.1. Definition

Clinical efficacy will be evaluated with the Clinical Assessment Tools. The investigator will use the Clinical Evaluation Tool to assess the severity of signs and symptoms with a total score from 0 to 18 at visit baseline, Day 7 and Day 14. The total scores at Day 7 and Day 14 compared with baseline scores will be used to define the percentage of clinical improvement.
A score from 1 to 5 will be assigned at Day 7 and at Day 14 based on the percentage of clinical improvement using Investigator Global Evaluation Tool.
Clinical Response is defined as having scores 1 or 2 ("healed" or "markedly improved").

5.2.2. Analysis Methods

The primary analysis will be performed on participants that have completed the 7-day treatment regime with prescribed itraconazole and had clinical evaluations at 7 days. Clinical Response score will be collected at Day 7. The number and percentage of subjects having score 1 or 2 ("healed" or "markedly improved") in Investigator Global Evaluation Tool will be summarized The point estimate and the corresponding 95% Confidence Interval for the proportion of participants who have clinical response will be provided using One-Proportion Z-test. Listing will be presented for all the subjects in efficacy analysis Set.

5.3. Major Secondary Endpoints

Major secondary endpoints include:

- Estimate the proportion of participants prescribed oral itraconazole who have mycological response after 14 days of follow-up
- Association of the clinical response at Day 7 with plasma drug concentrations of itraconazole and hydroxy-itraconazole
- Association of the clinical response at Day 7 with the baseline sensitivity pattern of causative fungi

- Estimation of the proportion of participants with clinical response after 14 days of follow-up. Estimate the extent to which clinically improved at Day 7 predicts clinically improved at Day 14.

5.3.1. Definition

- **Mycological response -** Mycological response is defined as both culture and microscopy result as negative.

- Plasma concentrations of itraconazole and hydroxy-itraconazole during therapy will be collected to measure the relationship between efficacy and plasma drug concentration.

- The Baseline minimum inhibitory concentration (MIC) is defined as the MIC of itraconazole toward fungal pathogens obtained prior to the administration of study drug.

- Clinical Response is defined as having scores 1 or 2 ("healed" or "markedly improved") based on the Investigator Global Evaluation tool of clinical improvement

5.3.2. Analysis Methods

- **Proportion of participants prescribed oral itraconazole who have mycological response after 14 days of follow-up**: To evaluate mycological cure skin scrapings collection for KOH mount and culture will be conducted at Baseline and at Day 14. Proportion of participants prescribed oral itraconazole who have mycological response after 14 days follow-up will be presented along with95% CI. A separate listing will be presented for each subject in efficacy analysis set.

- **Association of the clinical response at Day 7 with the baseline (MIC) sensitivity pattern of causative fungi**

  A logistic regression will be fitted to determine the association. The clinical response at Day 7 will be considered as the dependent variable, and baseline MIC as the independent regressor. In case of strong association, the baseline MIC value will be able to accurately predict the category of clinical response.

- **Association of the clinical response at Day 7 with plasma drug concentrations**: Plasma concentrations of itraconazole and hydroxy-itraconazole collected from venous blood samples will be assessed against clinical outcomes at Day 7. The relationship between clinical response and plasma drug concentrations at matching time points will be assessed graphically. Scatter plots for individual clinical response at each time point of measurement will be plotted against the corresponding plasma concentration.

- **Association of the clinical response at Day 7 with the baseline sensitivity pattern of causative fungi:** Proportion of subjects who have clinical response after 14 days of follow up will be calculated and 95% CI will be provided using One-Proportion Z-test.
  The ratio of clinical responders at Day7 to responders at Day14 will be calculated for estimating the extent to which clinically improved at Day 7 predicts clinically improved at Day 14.

6. SAFETY

All safety analyses will be based on the safety analysis set based on actual treatment received, unless otherwise specified.

For all continuous safety variables, descriptive statistics will include the N, mean, standard deviation, median, minimum, and maximum. Categorical variables will be summarized using frequency counts and percentages.

6.1. Adverse Events

The verbatim terms used in the CRF by investigators to identify adverse events will be coded using the available version of the Medical Dictionary for Regulatory Activities (MedDRA). Treatment-emergent adverse events are adverse events with onset during the treatment phase or that are a consequence of a pre-existing condition that has worsened since baseline. If the event occurs on the day of the initial administration of study agent, and either event time or time of administration are missing, then the event will be assumed to be treatment emergent. If the event date is recorded as partial or completely missing, then the event will be considered to be treatment emergent unless it is known to be prior to the first administration of study agent based on partial onset date or resolution date. All reported adverse events will be included in the analysis. For each adverse event, the number and percentage of subjects who experience at least 1 occurrence of the given event will be summarized by treatment.

Summary tables will be provided for:

- AEs
- Serious AEs (SAEs)
- AEs leading to discontinuation of treatment
- AEs by severity (Mild, Moderate, Severe)
- AEs by relationship to treatment (Not Related, Doubtful, Possible, Probable, Very Likely)

In addition to the summary tables, listings will be provided for subjects who:

- Had SAEs
- Had AEs leading to discontinuation of treatment
- Persistent AEs (AEs not resolved at EOS/Withdrawal/Follow-up visits).
- AEs Leading to Death

A listing of subjects who died will be provided.

6.2. Clinical Laboratory Tests and Vital Signs Findings

All clinical laboratory parameters and vital sign parameters including pulse, blood pressure (systolic and diastolic) will be listed by subjects.

7. PHARMACOKINETICS/PHARMACODYNAMICS

7.1. Pharmacokinetics

PK analyses will be performed on the PK analysis set, defined as participants that have at least one set of blood samples drawn at Day 7 to evaluate plasma drug concentrations of itraconazole and its main metabolite, hydroxy-itraconazole.

Blood sample collection will occur 24 hours (±2 hours) after previous dose (Day 6, Day 13) and 2 and 4.5 hours ±10 minutes after the last dose (Day 7, Day 14).

Data will be summarized for plasma concentration of itraconazole and hydroxy-itraconazole at each time point by treatment and listing will be provided for all participants with available plasma concentrations for itraconazole and reference itraconazole.

8. BIOMARKERS

Not applicable

9. HEALTH ECONOMICS

Not applicable

REFERENCES

1. Committee for Medicinal Products for Human Use. Guideline on the Investigation of Bioequivalence. January 2010.
2. U.S. Department of Health and Human Services, Food and Drug Administration. Guidance for Industry: Bioavailability and Bioequivalence Studies for Orally Administered Drug Products – General Considerations, March 2003.
3. Shankar G., Arkin S., Cocea L., Devanarayan V., Kirshner S., Kromminga A., quarmby V., Richardws,S, Schnieder C.K. Assessment and Reporting of the Clinical Immunogenicity of Therapeutic Proteins and Peptides – Harmonized Terminology and Tactical Recommendations. The AAPS Journal. e-ISSn 1550-7416. AAPS J DOI 10.1208/s12248-014-9599-2
