# Supplementary material for: No evidence of resistance to itraconazole in a prospective real-world trial of dermatomycosis in India
Source: PLoS One. 2023 Feb 14;18(2):e0281514. doi: 10.1371/journal.pone.0281514 (PMC9928099; doi:10.1371/journal.pone.0281514)
Supplement: S4 File — (DOCX) [file pone.0281514.s005.docx]

**Mycology testing methods:**

**Specimen collection:** A scraping of the skin (specimen) was taken with a sterile blunt scalpel from the edge of the lesion. The scrapings were placed inside Dermapak and sealed. A paperclip was used to prevent them from opening during shipping. Sealed dermapaks were placed inside the small primary plastic bag (which comes with each dermapak) and were then placed inside the secondary biohazard bag prior to shipping (more than 1 dermapak/plastic bag was placed inside the bag). The samples were directly shipped to the central laboratory.

**Identification:** The fungal pathogens were identified by amplification and Sanger-based (dideoxy‑termination) sequencing as per the Clinical and Laboratory Standards Institute (CLSI) MM18-A guidelines.

**Analysis:** The KOH samples were analyzed locally at each study center. The mycological culture analysis was centralized and performed in the University of Texas - Fungus Testing Lab - San Antonio, Texas, US.

**Analysis Protocol:** The Clinical and Laboratory Standards Institute (CLSI) M27 guidelines were followed for sample preparation and analysis.

**Quantitative Results: Microbial susceptibility**

| **Test name** | **Ketoconazole** | **Fluconazole** | **Itraconazole** | **Voriconazole** | **Terbinafine** | **Griseofulvin** |
| --- | --- | --- | --- | --- | --- | --- |
| **LLOQ, mcg/mL** | **0.03** | **0.125** | **0.03** | **0.03** | **0.004** | **0.03** |
| **ULOQ, mcg/mL** | **16** | **64** | **16** | **16** | **2** | **16** |

LLOQ, lower limit of quantification; ULOQ, upper limit of quantification
